# Enhanced rates photocatalytic degradation of acid red 37 dye by TiO_2_–SnO_2_ in solid state under UV-irradiation light

**DOI:** 10.1186/s11671-025-04397-2

**Published:** 2025-12-18

**Authors:** Wagih Sadik, Abdelghaffar Eldemerdash, Adel William Nashed, Amr Ahmed Mostafa, Elsayed Lamie

**Affiliations:** Materials Science Department, Institute of Graduate Studies and Research, Alexandria University, Alexanderia, Egypt

**Keywords:** Photocatalysis, Solid state, TiO_2_/SnO_2_ nanocomposite, Wastewater treatment, UV visible. Inorganic antioxidant, Organic compounds

## Abstract

A variety of compounds have been treated with lit semiconductors for water pollution treatment. UV irradiation with oxidants or catalysts (TiO_2_) can remediate industrial effluent. The goal of this study is to prepare and characterize TiO_2_–SnO_2_ nanocomposites with different ratios to degradation dyes in wastewater treatment. These nanocomposites will be used to photocatalysis degradation of acid red 37 dye in aqueous solution using UV irradiation as a model pollutant. The characterization of TiO_2_–SnO_2_ nanocomposites was conducted using XRD, TEM, EDAX, FTIR, and XPS. The photocatalytic degradation was tested with several advanced oxidation processes (AOPs) by adding varying concentrations of SnO_2_ (0–20 wt.%), and then by employing an inorganic antioxidant (H_2_O_2_, Na_2_S_2_O_8_, NaIO_4_) with a ratio of UV/TiO_2_–SnO_2_ (90:10) to high efficiency of degradation of acid red 37 dye using a batch photoreactor. Consequently, the effects of pH, dye concentrations, and different loading (g/L) of nanocomposite on the photocatalytic activity of the SnO_2_–TiO_2_ nanocrystals were examined. The UV/TiO_2_–SnO_2_ (90:10) wt.% has higher photocatalytic efficiency for degradation acid red 37 dye from either TiO_2_, SnO_2_ or the other ratios in this study.

## Introduction

The generation of clean and sustainable energy, together with environmental issues, has emerged as the foremost challenge confronting humanity. The global population growth and the economic development over the past decade are persistently accelerating. The exponential growth of the population, the expansion of the industrial, and the agricultural productions have led to an increasing demand for the planet's finite freshwater supplies. In this context, the conservation of natural water resources and the development of innovative technologies for water purification and wastewater management have emerged as key environmental issues of the twenty-first century.

Water pollution, especially from organic contaminants, has emerged as one of society's most significant concerns due to industrialization. The carcinogenic and the teratogenic effects of organic contaminants jeopardize human health [1]. Moreover, organic pollutants can deplete substantial amounts of dissolved oxygen which lead to impair both normal development of aquatic organisms and the deterioration of water quality [2, 3].

During the dyeing process, between 1 and 20 wt.% of total world dye output is wasted and discharged into the environment as textile effluent. Because of these compounds' possible carcinogenic qualities, the environmental effect of these dyes is a big dilemma. Furthermore, the weathering of these organic dyes in the wastewater phase by chemical reactions such as oxidation, hydrolysis, anaerobic discoloration, or others might yield possible carcinogens that are harmful to the health of animals and human beings, as well. These dyes can reduce the amount of oxygen that dissolves in colored wastewater which is necessary for aquatic life. As a result, it is imperative to adequately cleanse these colored effluents before discharging them into various bodies of water [4].

The traditional methods to treat these effluents are by splitting biochemical, chemical, and physical components. Traditional methods of wastewater treatment are ineffective since they partially remove colors or are ineffective on contaminants that are neither volatile nor easily adsorbable. They also have extra drawbacks since they just move pollutants to another phase, causing harmful disposal issues. Biological treatment necessitates a huge amount of land and is limited by diurnal sensitivity, the toxicity of some chemicals, and a lack of design, and operating flexibility. Many organic compounds can be destroyed in this manner, while many others are in resistance [5].

For a semiconductor to exhibit photochemical activity, its redox potential must be sufficiently positive to enable the photogenerated valence band hole to generate adsorbed OH radicals that can be capable of oxidizing organic pollutants. In the meantime, to be sufficiently negative to allow the conduction band electron to reduce the adsorbed O_2_ to the superoxide [6].

Catalysts play a crucial part in the photocatalytic process. Titanium dioxide TiO_2_, among the many photocatalysts, is crucial due to its lack of toxicity, unique physical characteristics, and chemical stability. It is often used in the production of hydrogen, the conversion of energy, and the treatment of wastewater [7, 8]. Catalysts are essential in the photocatalytic process which will essentially and necessarily enhance the photocatalytic activity of the modified titanium dioxide. Titanium-based composites have been investigated with precious metals, transition metals, non-metallic elements, metal ions, and rare earth metal ion doping as effective means for enhancing photocatalytic activity [9–11]. When photons interact with semiconductor materials, a catalytic process known as photocatalysis occurs on their surface. Developing photocatalysts that enhance charge carrier separation and facilitate commercial applications for environmental remediation and hydrogen production is a significant challenge for researchers in the field of photocatalysis [12].

Photocatalysis, due to its numerous benefits, such as its simplicity of use, high efficiency, and lack of secondary pollutants, has received a lot of attention as one of the (AOPs) in recent years. Titanium dioxide (TiO_2_) is noteworthy among photocatalysts due to its non-toxic nature, distinctive physical properties, and chemical stability. It is frequently utilized in hydrogen production, energy conversion, and wastewater treatment. TiO_2_ acts as a medium when light is stimulated and acts as a semiconductor, allowing electrons to flow from the valence band to the conduction band, while holes (h^+^) stay in the valence band. As a result, electrons and holes move to the semiconductor surface and engage in a series of reduction and oxidation reactions. Consequently, electrons and holes migrate to the semiconductor surface and participate in a sequence of reduction and oxidation reactions [13].

**Fig. 1:**
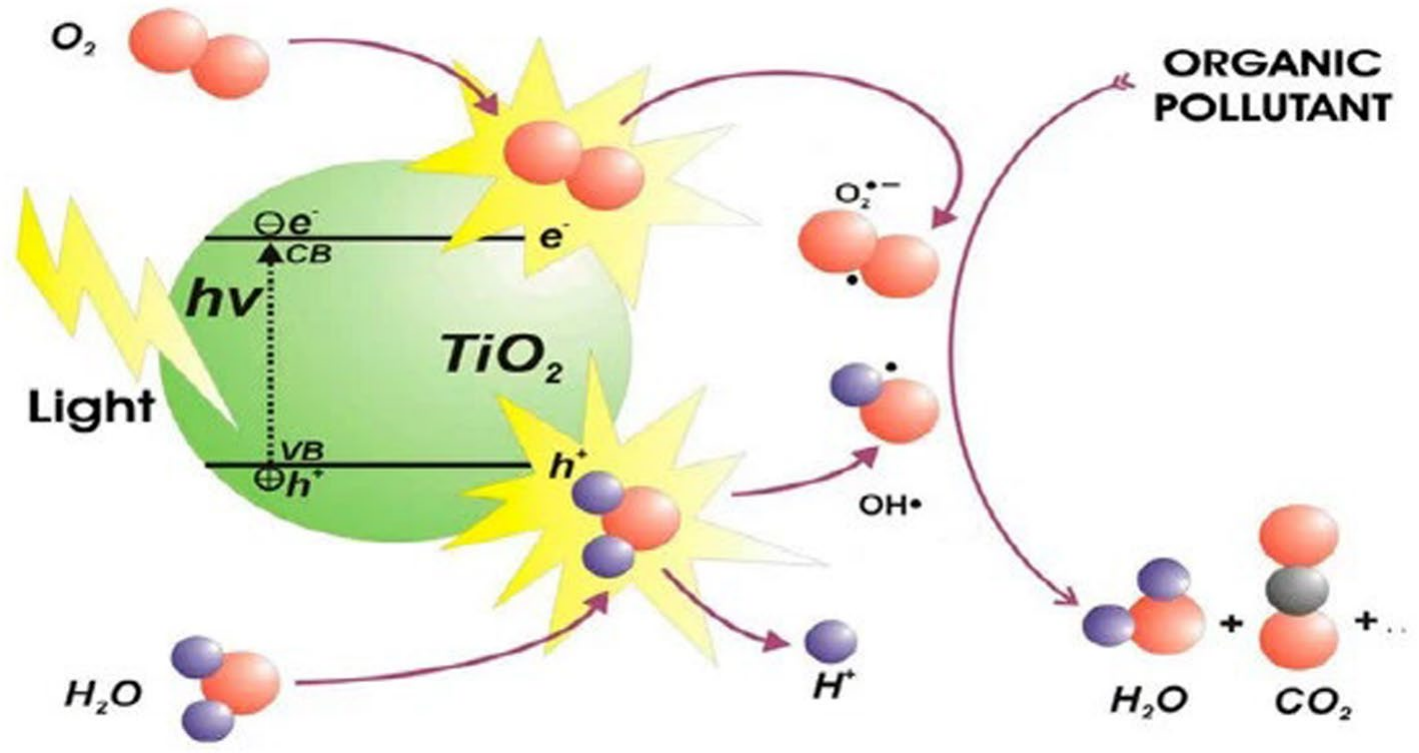
Schematic representation of the basic principles of photodegradation process using semiconductors (example: TiO_2_)

TiO_2_ is a component of metal-oxide semiconductor photocatalysts. TiO_2_ serves as an effective n-type semiconductor photocatalyst due to its high activity, durability against photo and chemical corrosion under reaction conditions, strong oxidizing capability, stability, and non-toxicity. TiO_2_ nanostructures represent a promising semiconductor for diverse applications, such as batteries, solar cells, dye pollution catalysis, hydrogen generation, and water splitting [14, 15] (Fig. 1).

Tin oxide (SnO_2_) has gained a significant amount of scientific attention due to its exceptional physical and chemical characteristics. At room temperature, SnO_2_ is an exceptional optical and electrical material that is an n-type semiconductor with a wide band gap (3.6 eV), low resistivity, and a high theoretical specific capacity. The electron mobility in SnO_2_ is quite high, ranging from 100 to 200 cm^2^ V^1^s^1^, which implies photoexcited electron transport occurs at a faster pace. SnO_2_ has been examined as a material that is promising due to its unique surface characteristics which include photocatalytic activity and luminescence. High band gap energies, high stability, and unique optical and structural properties are all exhibited by SnO_2_ nanoparticles [16].

## Results and discussion

### Characterization of the prepared TiO_2_–SnO_2_ nanocomposites

**Fig. 2:**
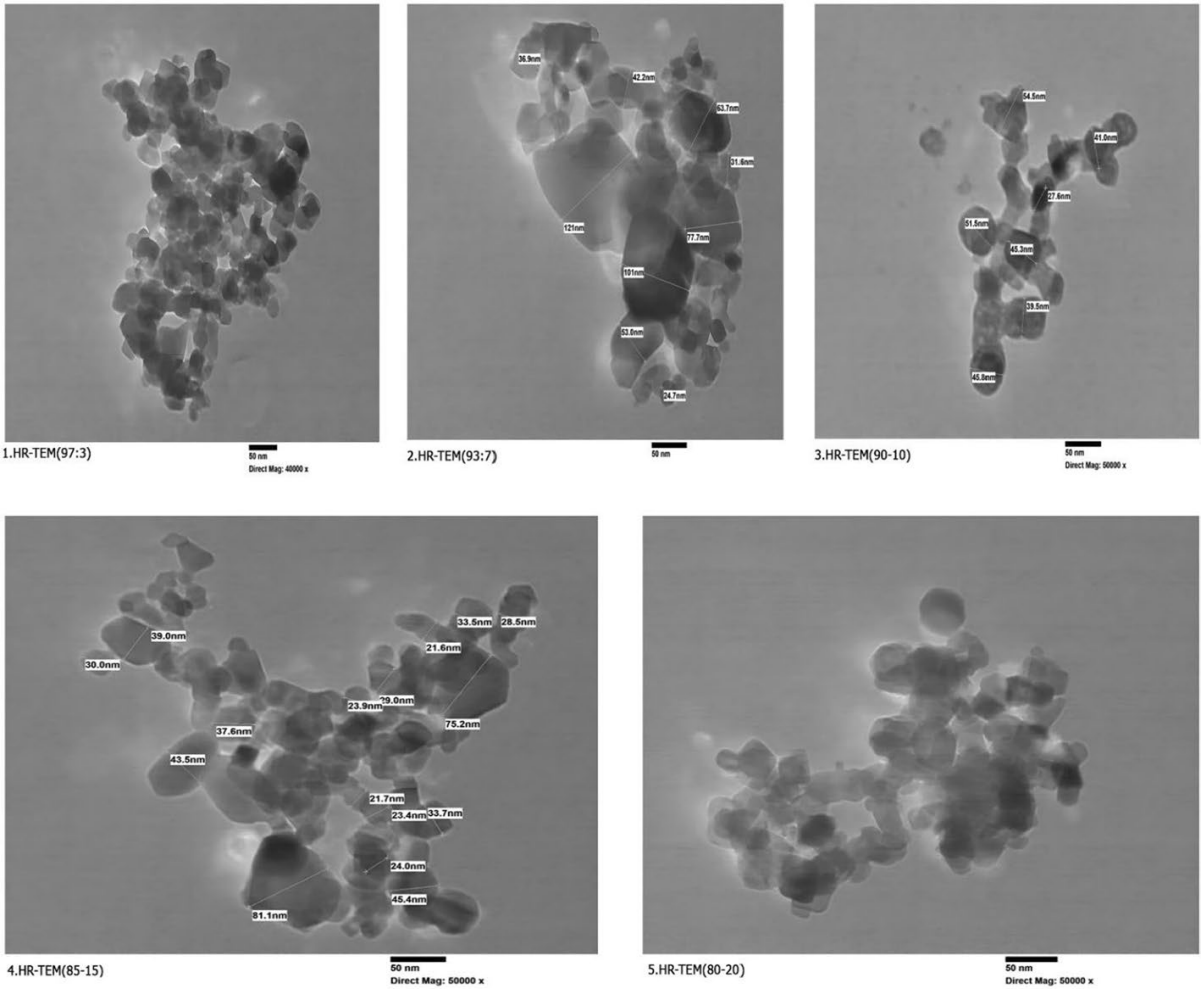
HR-TEM of TiO_2_–SnO_2_ (97:3) wt.% nanocomposite. **a** TiO_2_–SnO_2_ (97:3) wt.% nanocomposite. **b** TiO_2_–SnO_2_ (93:7) wt.% nanocomposite. **c** TiO_2_–SnO_2_ (90:10) wt.% nanocomposite. **d** TiO_2_–SnO_2_ (85:15) wt.% nanocomposite. **e** TiO_2_–SnO_2_ (80:20) wt.% nanocomposite

Figure 2 shows the HR-TEM pictures as indicated in (a) of the synthesized TiO_2_–SnO_2_ (97:3) wt.% nanocomposite. The particles had tetragonal shape with a diameter of 26.3–88 nm, according to a detailed analysis of the micrograph.

The TEM image of the synthesized TiO_2_–SnO_2_ (93:7) wt.% nanocomposite is depicted in Fig. 2b. According to a comprehensive examination of the micrograph, the particles were tetragonal in shape and had a diameter ranging from 26.6 to 64.4 nm. The TEM image of the synthesized TiO_2_–SnO_2_ (90:10) wt.% nanocomposite is shown in Fig. 2c. According to a comprehensive examination of the micrograph, the particles were approximately tetragonal and semi-round in shape with a diameter ranging from 27.6 to 54.5 nm.

The HR-TEM image of the synthesized TiO_2_–SnO_2_ (85:15) wt.% nanocomposite is displayed in Fig. 2d. The particles were characterized by a tetragonal shape and a diameter of 28.4–72 nm, as determined by a comprehensive micrograph analysis.

The prepared TiO_2_–SnO_2_ (80:20) wt.% nanocomposite is illustrated in Fig. 2e using a transmission electron microscope. The particles were tetragonal and semi-round in shape, with a diameter ranging from 30.2 to 72.2 nm, as indicated by a comprehensive micrograph analysis.

**Fig. 3:**
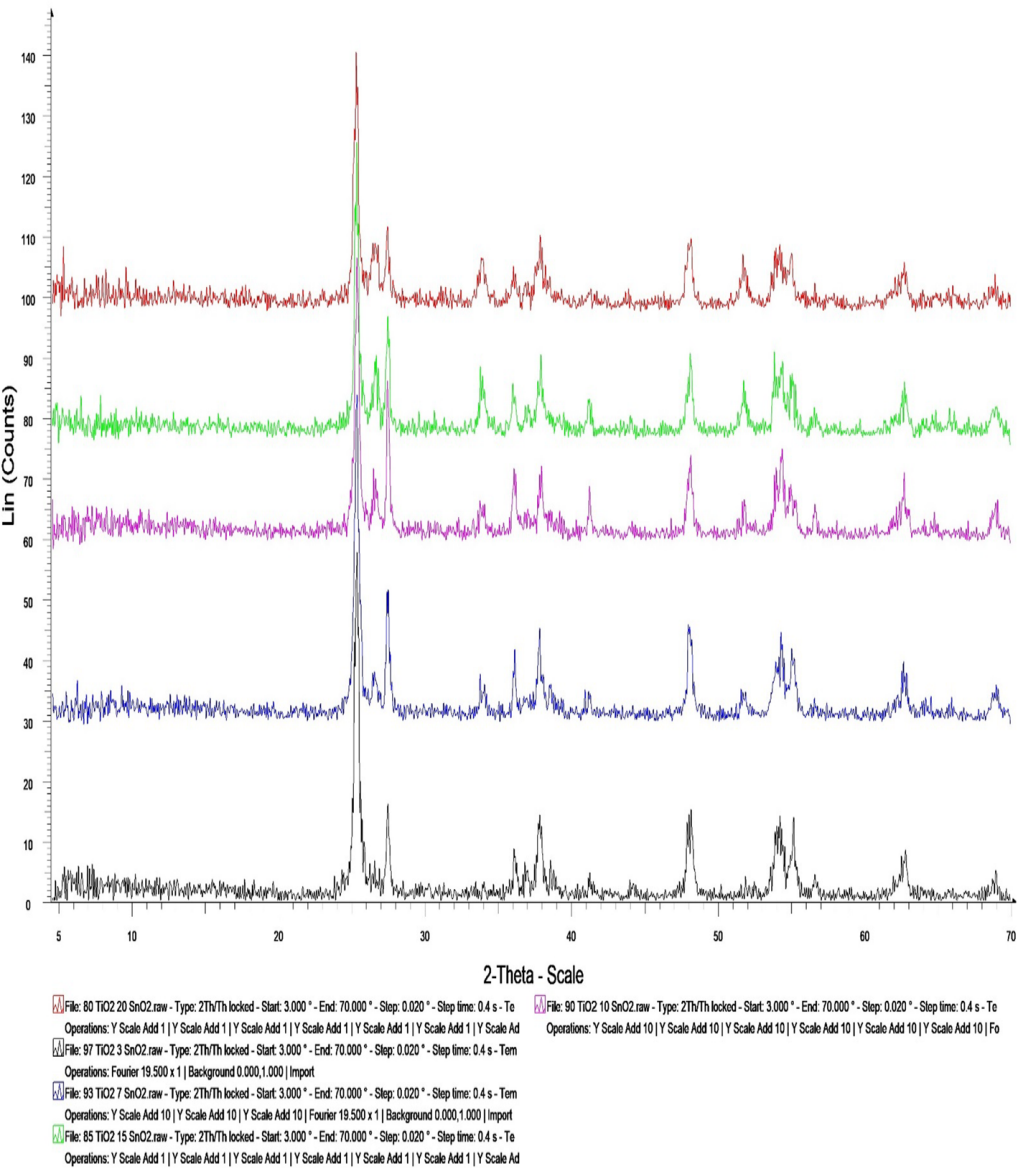
XRD of TiO_2_–SnO_2_ (0–20) wt.% nanocomposites

**Fig. 4:**
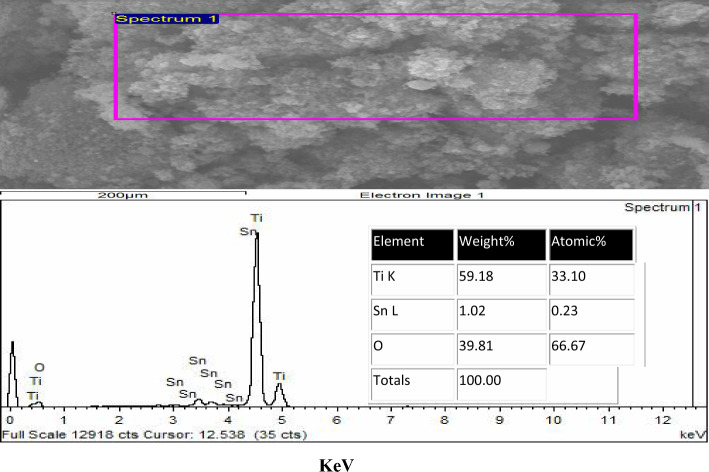
EDAX of TiO_2_–SnO_2_ (97:3) wt.% nanocomposite

**Fig. 5:**
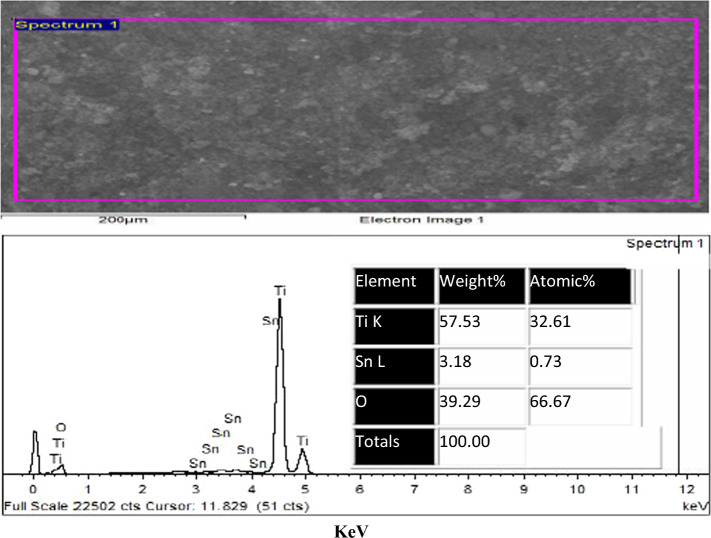
EDAX of TiO_2_–SnO_2_ (93:7) wt.% nanocomposite

**Fig. 6:**
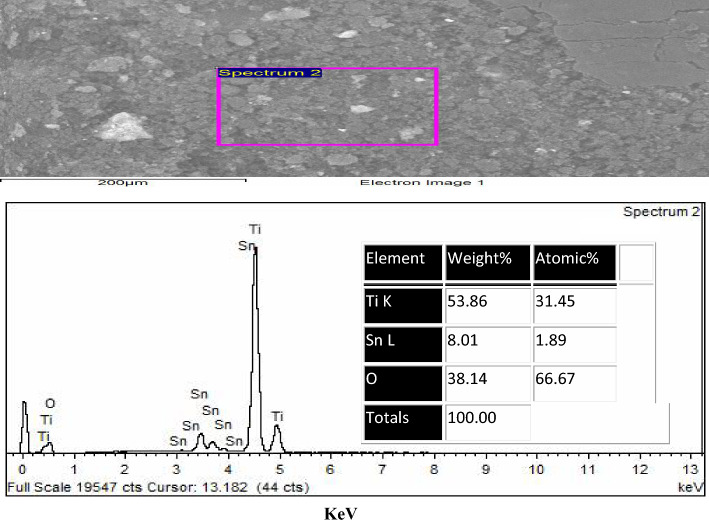
EDAX of TiO_2_–SnO_2_ (90:10) wt.% nanocomposite

It is evident from the preceding Figs. 2, 3, 4, 5, 6 that the particle size increases as the concentration of SnO_2_ (3–7 wt.%) in the TiO_2_–SnO_2_ nanocomposite increases. The particle size of the nanocomposite increases as the SnO_2_ concentration increases to 10 wt.%, while the particle size of the nanocomposite increases as the SnO_2_ concentration increases to 20 wt.% due to agglomeration.

### XRD of the prepared TiO_2_–SnO_2_ nanocomposites

XRD analysis is employed to evaluate the purity, crystallite size, and crystal structure of the nanoparticles that are produced. Crystallite size is determined by the breadth of a specific XRD peak in a diffraction pattern that is associated with a specific planar reflection within the crystal unit cell. The peak broadening reflects the crystallites' size. Having a standard card issued by the Joint Committee of Powder Diffraction Standards (JCPDS card No 041-1445 and 001-0562), the measured diffraction peaks are rendered to be consistent.

Figure 3 is represented XRD for different ratios such as:

**TiO**_**2**_**–SnO**_**2**_** (97:3) wt.% nanocomposite sample** The XRD pattern of the TiO_2_–SnO_2_ (97:3) wt.% nanocomposite sample is illustrated with diffraction peaks at 2θ = 25.30, 26.56, 27.42, 33.92, 36.09, 37.81, 38.65, 41.29, 47.99, 51.85, 54.03, 55.0, and 68.92. The crystal tetragonal structure's allocated pattern which indicates how it was formed, corresponds to TiO_2_–SnO_2_ (97:3) wt.%. This structure exhibited a modest peak shift and an increase in peak intensity, with an average crystal size of 42.3 nm.

**TiO**_**2**_**–SnO**_**2**_** (93:7) wt.% nanocomposite sample** illustrates the XRD pattern of the TiO_2_–SnO_2_ (93:7) wt.% nanocomposite sample, which exhibits diffraction peaks at 2θ = 25.29, 26.51, 27.41, 33.77, 36.08, 37.79, 41.13, 47.99, 51.70, 54.34, 55.07, 62.77, and 68.92. The tetragonal crystal structure's allocated pattern, which indicates how it was formed, corresponds to TiO_2_–SnO_2_ (93:7) wt.%. This structure exhibited a modest peak shift and an increase in peak intensity, with an average crystal size of 41.1 nm.

**TiO**_**2**_**–SnO**_**2**_** (90:10) wt.% nanocomposite sample** illustrates the XRD pattern of the TiO_2_–SnO_2_ (90:10) wt.% nanocomposite as the sample is showing, with diffraction peaks at 2θ = 25.33, 26.66, 27.43, 33.82, 36.08, 37.92, 41.24, 48.08, 51.75, 54.33, 56.68, 62.72, and 68.92. The tetragonal crystal structure's allocated pattern, which indicates how it was formed, corresponds to TiO_2_–SnO_2_ (90:10) wt.%. This structure exhibited a modest peak shift and an increase in peak intensity, with an average crystal size of 37.09 nm.

**TiO**_**2**_**–SnO**_**2**_** (85:15) wt.% nanocomposite sample** Illustrates the XRD pattern of the TiO2–SnO2 (85:15) wt.% nanocomposite sample, as exhibiting diffraction peaks at 2θ = 25.29, 26.56, 27.43, 33.83, 36.08, 35.92, 37.9, 41.14, 48.11, 51.66, 54.9, 55.06, and 68.88. The tetragonal crystal structure's designated pattern, indicative of its formation, corresponds to TiO_2_–SnO_2_ (85:15) wt.%. This exhibited a minor peak shift and an enhancement in peak intensity, with an average crystal size of 40.5 nm.

**TiO**_**2**_**–SnO**_**2**_** (80:20) wt.% nanocomposite sample** Illustrates the XRD pattern of the TiO_2_–SnO_2_ (80:20) wt.% nanocomposite sample, as exhibiting diffraction peaks at 2θ = 25.28, 26.57, 27.39, 33.87, 36.92, 35.92, 37.78, 41.29, 48.09, 51.70, 54.14, 62.77, and 68.86. The tetragonal crystal structure's designated pattern, indicative of its formation, corresponds to TiO_2_–SnO_2_ (80:20) wt.%. It exhibited a little peak shift and an enhancement in peak intensity, with an average crystal size of 38.5 nm.

The XRD results of TiO_2_–SnO_2_ nanocomposites indicate that the average crystal size of TiO_2_–SnO_2_ (97:3) wt.% was 42.3 nm, which decreased to 41.1 nm and 37.05 nm when the weight percentage of SnO_2_ increased from 7 to 10%. The increasing of the SnO_2_ weight percentage to 15% and 20% led to a reduction in the average crystal size to 40.9 nm and 38.5 nm, respectively, as indicated in Table 1.

**Table 1:** Average crystal size (nm) of the prepared TiO_2_–SnO_2_ nanocomposites

TiO_2_–SnO_2_ nanocomposite (wt.%)	Average crystal size (nm)
(97:3)	42.3
(93:7)	41.1
**(90:10)**	**37.05**
(85:15)	40.9
(80:20)	38.5

### EDAX of the prepared TiO_2_–SnO_2_ nanocomposite

#### EDAX of TiO_2_–SnO_2_ (97:3) wt.% nanocomposite

Figure 4 illustrates the EDAX of the TiO_2_–SnO_2_ (97:3) wt.% nanocomposite. The spectrum indicates a pronounced Ti signal at around 4.5 eV and a subtle signal at roughly 0.45 eV, with an atomic percentage of 33.1. A weak oxygen signal, with an atomic percentage of 66.67, was observed at around 0.45 eV. Two weak signals for tin were detected at around 0.94 and 8.04 eV, corresponding to an atomic percentage of 0.23.

#### EDAX of TiO_2_–SnO_2_ (93:7) wt.% nanocomposite

Figure 5 depicts the EDAX of the synthesized TiO_2_–SnO_2_ (93:7) wt.% nanocomposite. The spectrum reveals a prominent Ti signal at roughly 4.5 eV and a muted signal around 0.45 eV, corresponding to an atomic percentage of 32.61. An oxygen signal was detected at around 0.45 eV, corresponding to an atomic percentage of 66.67. Two faint signals for tin were identified at around 0.94 and 8.04 eV, corresponding to an atomic percentage of 0.73.

#### EDAX of TiO_2_–SnO_2_ (90:10) wt.% nanocomposite

Figure 6 shows the EDAX of the synthesized TiO_2_–SnO_2_ (90:10) wt.% nanocomposite. The spectrum exhibits a strong Ti signal at around 4.5 eV and a faint signal at about 0.45 eV with an atomic percentage of 31.45. There was also a modest signal for oxygen at roughly 0.45 eV with an atomic percentage of 66.67. Two faint signals for tin were recorded at around 0.94 and 8.04 eV with an atomic percentage of 1.89.

#### EDAX of TiO_2_–SnO_2_ (85:15) wt.% nanocomposite

**Fig. 7:**
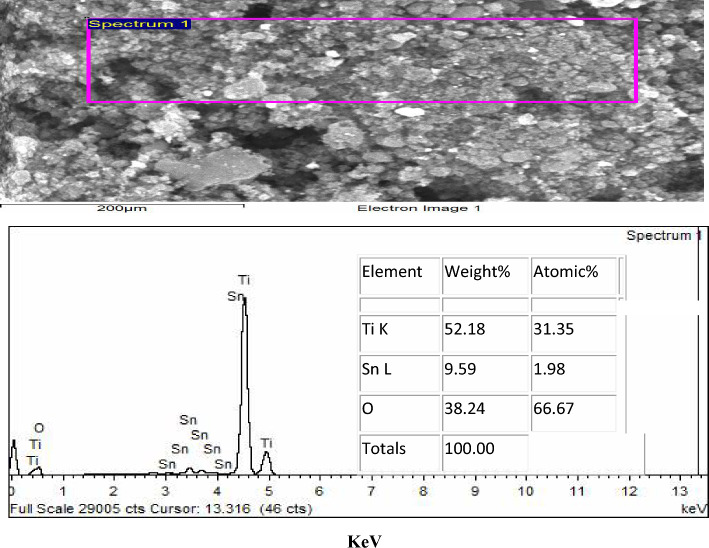
EDAX of TiO_2_–SnO_2_ (85:15) wt.% nanocomposite

Figure 7 illustrates the EDAX of the produced TiO_2_–SnO_2_ (85:15) wt.% nanocomposite. The spectrum indicates a prominent Ti signal at approximately 4.5 eV and a subtle signal near 0.45 eV, with an atomic percentage of 31.35. A small signal for oxygen was observed at approximately 0.45 eV, with an atomic percentage of 66.67. Two weak signals for tin were detected at around 0.94 and 8.04 eV, with an atomic percentage of 1.98.

#### EDAX of TiO_2_–SnO_2_ (80:20) wt.% nanocomposite

**Fig. 8:**
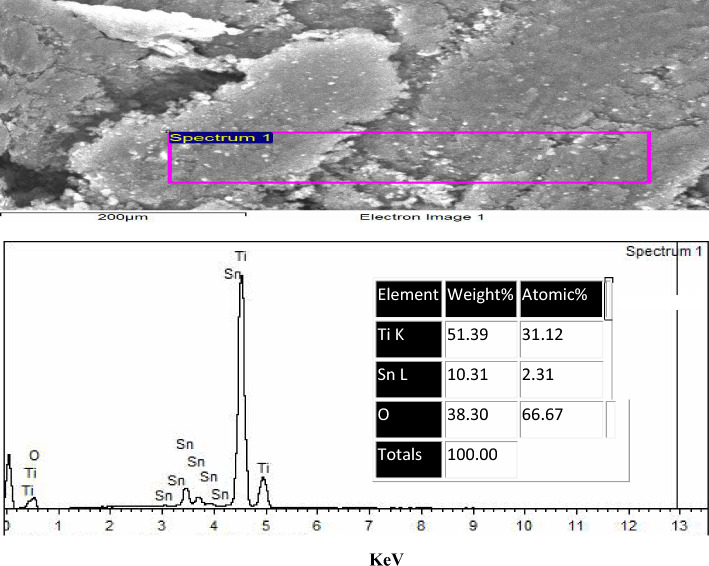
EDAX of SnO_2_–TiO_2_ (80:20) wt.% nanocomposite

Figure 8 shows the EDAX of the synthesized TiO_2_–SnO_2_ (80:20) wt.% nanocomposite. The spectrum reveals a strong Ti signal at around 4.5 eV and a faint signal at about 0.45 eV with an atomic percentage of 31.12. There was also a modest signal for oxygen at roughly 0.45 eV with an atomic percentage of 66.67. Two faint signals for tin were recorded at around 0.94 and 8.04 eV with an atomic percentage of 2.31.

The results obtained from the EDAX analysis of TiO_2_–SnO_2_ nanocomposites, illustrated in Figs. 4, 5, 6, 7, 8, validated the elemental ratios of the synthesized nanocomposites.

### FTIR of the prepared SnO_2_–TiO_2_ nanocomposites

#### FTIR of TiO_2_–SnO_2_ (97:3) wt.% nanocomposite

**Fig. 9:**
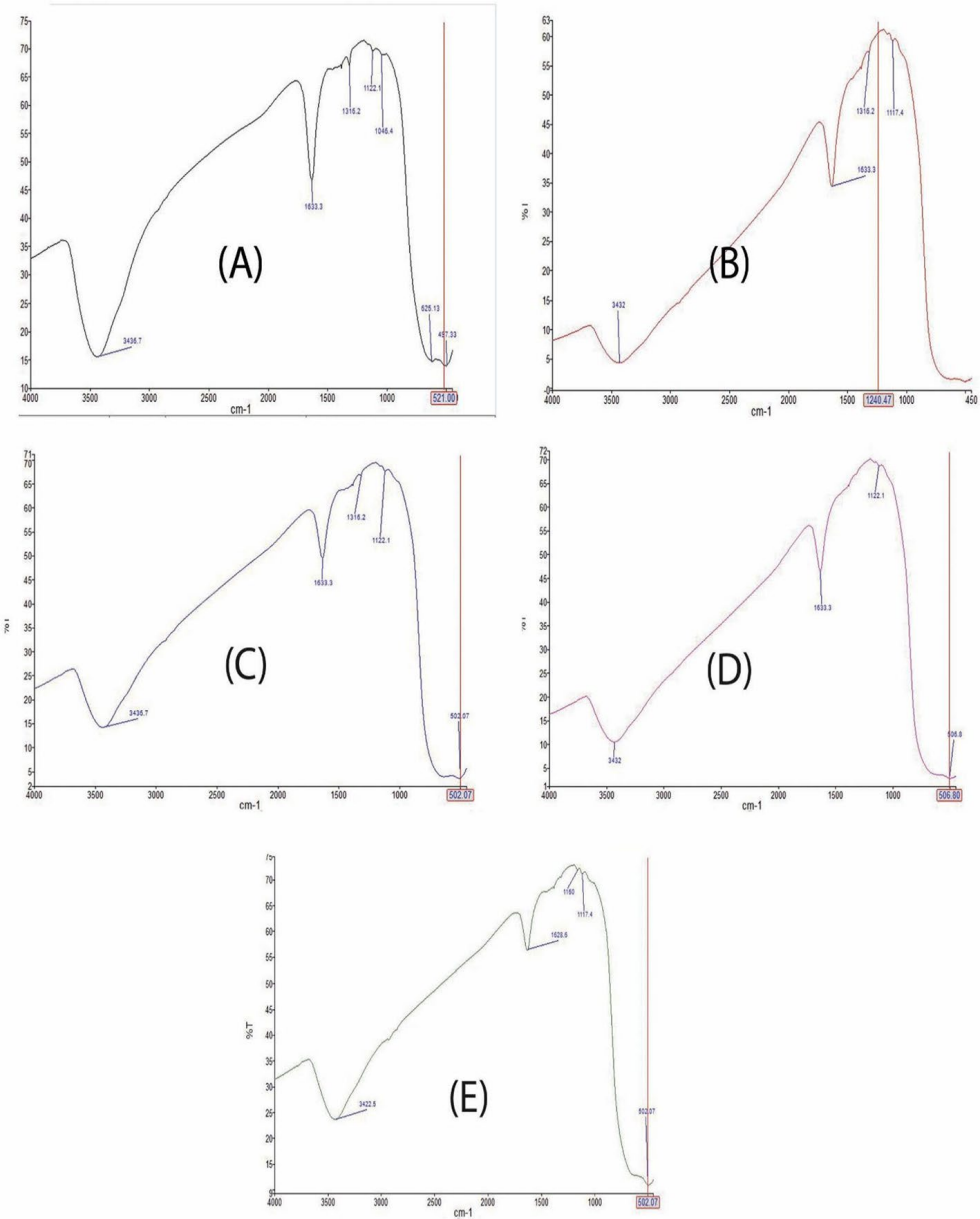
**A** FTIR of TiO_2_–SnO_2_ (97:3) wt.% nanocomposite. **B** FTIR of TiO_2_–SnO_2_ (93:7) wt.% nanocomposite. **C** FTIR of TiO_2_–SnO_2_ (90:10) wt.% nanocomposite. **D** FTIR of TiO_2_–SnO_2_ (85:15) wt.% nanocomposite. **E** FTIR of TiO_2_–SnO_2_ (80:20) wt.% nanocomposite

Figure 9A presents the FTIR spectra of the TiO_2_–SnO_2_ (97:3) wt.% nanocomposite, exhibiting a wide band at about 3436 cm^−1^, characteristic of non-hydrogen bound hydroxyl groups. The C–H bond accounts for the peak at 1316 cm^−1^, while the bending mode of adsorbed water corresponds to the prominent peak at 1633 cm^−1^. Ti–O and Sn–O display distinct vibrational frequencies at 625 and 497 cm^−1^, respectively.

**The FTIR spectra of the TiO**_**2**_**–SnO**_**2**_** nanocomposite (93:7) wt.%** was analyzed. Figure 9B displays a broad band at 3432 cm^−1^, characteristic of non-hydrogen bound hydroxyl groups. The C–H bond accounts for the peak at 1316 cm^−1^, while the bending mode of adsorbed water corresponds to the prominent peak at 1633 cm^−1^. The characteristic vibrations of inorganic Ti–O and Sn–O are observed at 700 and 450 cm^−1^, respectively.

**The FTIR spectra of the TiO**_**2**_**–SnO**_**2**_** (90:10) wt.%** nanocomposite in Fig. 9C exhibits a wide band at 3436 cm^−1^, characteristic of non-hydrogen-bound hydroxyl groups. The C–H bond accounts for the peak at 1316 cm^−1^, while the bending mode of adsorbed water corresponds to the prominent peak at 1633 cm^−1^. The inorganic Ti–O and Sn–O display unique vibrational frequencies at 700 and 502 cm^−1^, respectively.

The FTIR spectrum of the TiO_2_–SnO_2_ (85:15) wt.% Fig. 9B illustrates a prominent band at 3432 cm^−1^ in nanocomposite, indicative of non-hydrogen-bound hydroxyl groups. The C–H bond accounts for the peak at 1122 cm^−1^, while the bending mode of adsorbed water corresponds to the prominent peak at 1633 cm^−1^. The inorganic Ti–O and Sn–O display unique vibrational frequencies at 700 and 506 cm^−1^, respectively.

### FTIR of TiO_2_–SnO_2_ (80:20) wt.% nanocomposite

The FTIR spectra of the TiO_2_–SnO_2_ (80:20) wt.% nanocomposite Fig. 9E exhibits a wide band at 3422 cm^−1^, characteristic of non-hydrogen-bound hydroxyl groups. The C–H bond accounts for the peak at 1160 cm^−1^, while the bending mode of adsorbed water is responsible for the prominent peak at 1628 cm^−1^. The inorganic Ti–O and Sn–O display distinct vibrational frequencies at 700 and 502 cm^−1^, respectively.

Figure 9A–E illustrates the FTIR spectra of TiO_2_–SnO_2_ nanocomposites which exhibit modest shifts in the peaks of TiO_2_ and SnO_2_ as being attributable to variations in SnO_2_ loading percentages.

### XPS of the prepared TiO_2_–SnO_2_ nanocomposite

The X-ray photoelectron spectroscopy (XPS), or the electron spectroscopy for chemical analysis (ESCA), is an analytical technique that uses x-rays to irradiate a sample and subsequently examines the energy of the emitted electrons.

### XPS of TiO_2_–SnO_2_ (90:10) wt.% nanocomposite

**Fig. 10:**
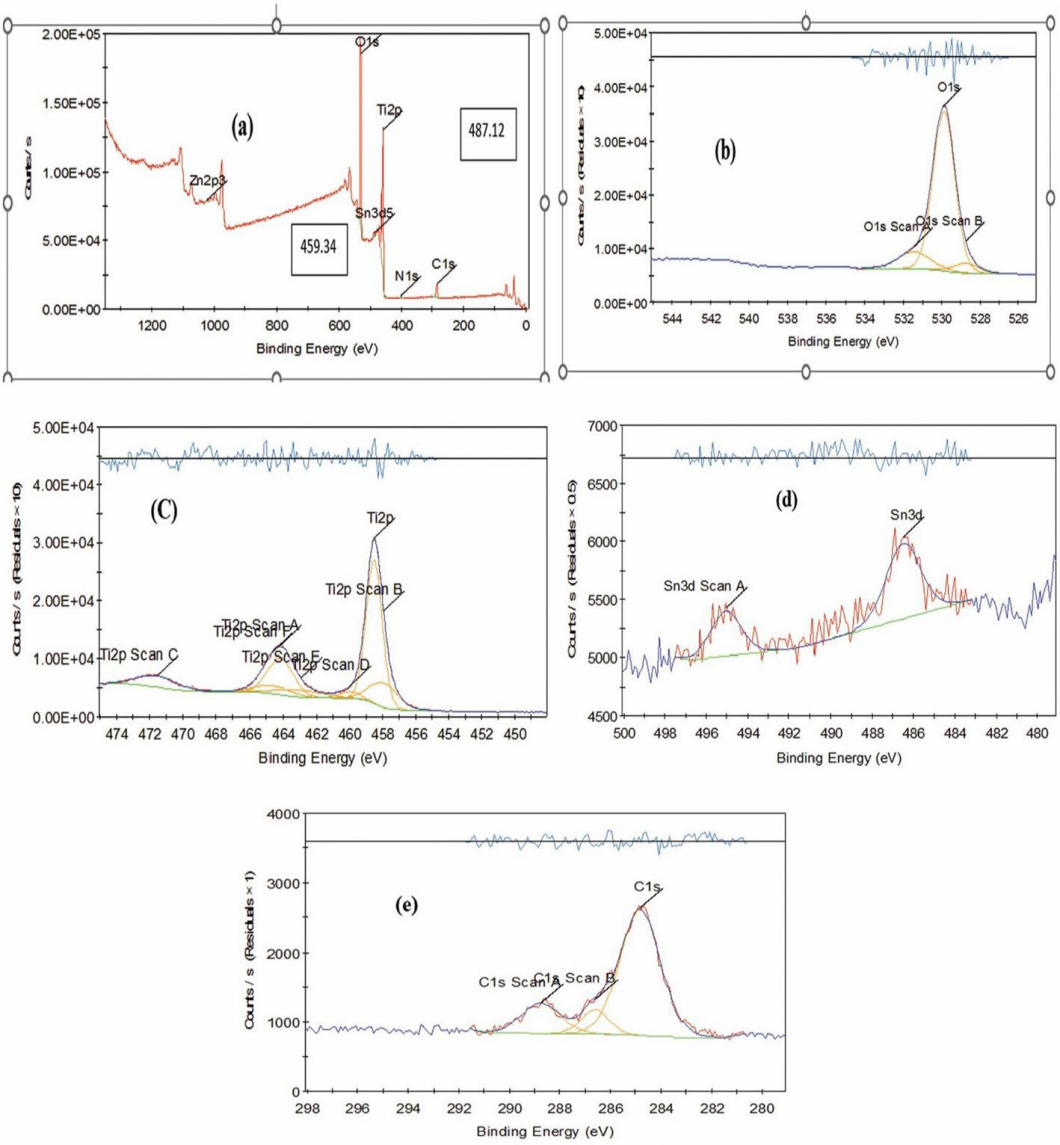
XPS of TiO_2_–SnO_2_ (90:10) wt.% nanocomposite calcined at 500 °C for 3 h. **a** Survey spectra; **b** O 1 s region; **c** Ti 2p region; **d** Sn 3d region; **e** C 1 s region

Figure 10 represents the outcomes of XPS investigation about the elemental composition and concentration. Peaks corresponding to O 1s, Ti 2p, Sn 3d, and C 1s were observed in the survey spectra Fig. 10a within the energy range of 0–1200 eV to indicate that Ti and O constituted the predominant elements in the samples. Figure illustratesthe spectra of asymmetric SnO_2_–TiO_2_ (90:10) as corresponding to the O 1s core level. The spectra were modeled using two Gaussian peaks at around 531.44 eV (Ti–O–Sn) and 528.75 eV (Sn–O). Despite the presence of residual Tin ions on the TiO_2_ surface, it was postulated that Ti–O–Sn bonds had predominantly been formed within the composite sample. It led to the elevation of the oxygen concentration at the surface (100). Six peaks in the Ti 2p area of the spectra Fig. 10c can be identified at energies of 464.15, 457.94, 471.54, 459.98, 462.91, and 464.69 eV. The peaks at 464.15 eV and 457.94 eV were ascribed to _2_/2p_1_^+^Ti^4^ and, _2_/2p_3_^+^Ti^4^, respectively. Figure 10d illustrates the doublet Sn 3d spectrum by exhibiting binding energies of 495.03 eV and 486.49 eV for the Sn^3+^3d_3/2_ and Sn3^+3^d_5/2_ lines, respectively [17].

Ultimately, the above Figure suggests that the catalysts may possess a greater number of lattice flaws. The presence of Sn^3+^ may facilitate the capture of electrons. The probability of recombination between photoinduced electrons and holes was decreased [17].

### Calculations of band gap energy of TiO_2_–SnO_2_ nanocomposite

**Fig. 11:**
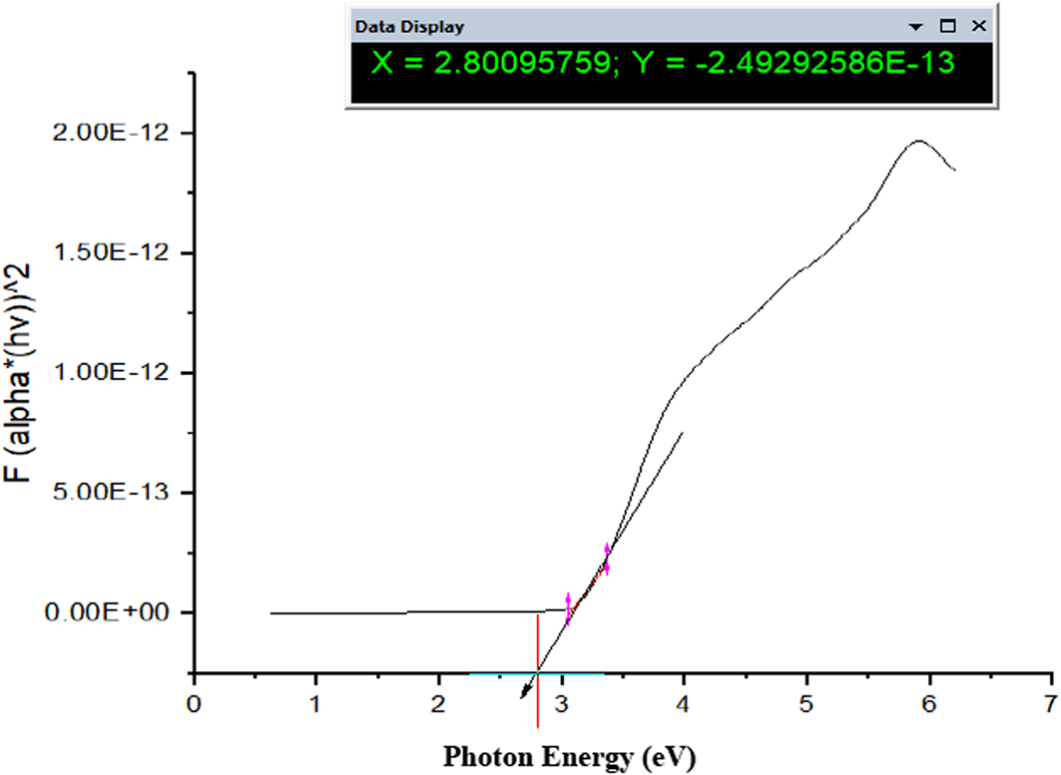
The band gap energy of TiO_2_–SnO_2_ (90:10) wt.% nanocomposite

Figure 11 illustrates the UV–vis spectrum of TiO_2_–SnO_2_ nanocomposites at a weight ratio of 90:10. Interband electronic transitions result in significant absorption below 400 nm in the material [8, 18]. Another absorption threshold may be located in the visible spectrum below 570 nm. SnO_2_ content rises, two absorption bands exhibit a substantial enhancement. Consequently, augmenting the concentration of SnO_2_ in the system induces a red shift towards longer wavelengths. This pertains to the emergence of novel energy levels within the band gap of TiO_2_ [19, 20]. Oxygen vacancies are the primary source of the minor absorption edges in the visible spectrum [21]. The band gap energy was computed using the Kubelka–Munk formula, as presented in Eq. (1).

1 $$ \alpha {\text{h}}\upsilon = {\text{A}}\left( {{\text{h}}\upsilon - {\text{Eg}}} \right)^{{\text{n}}} $$

where n = 1/2 for direct band gap materials and n = 2 for indirect band gap materials, α is the absorption coefficient, h is the Planck constant, υ is the wavenumber, A is a constant, and E_g_ is the band gap energy [22].

### Calculations of band gap energy of TiO_2_–SnO_2_ (90:10) wt.% nanocomposite

Figure 11 illustrates the band gap energies (Eg) of the TiO_2_–SnO_2_ (90:10) wt.% nanocomposite. As the weight percentage of SnO_2_ increases, two absorption bands exhibit a significant enhancement. The band gap energy (Eg) of TiO_2_–SnO_2_ (93:7) wt.% is ascertained from the graph of (hυ)^2^ versus photon energy in electron volts. The band gap energy of TiO_2_–SnO_2_ (97:3) wt.% nanocatalysts (2.8 eV) decreased as the weight percentage of SnO_2_ increased due to the charge transition between Sn ions and the conduction band of TiO_2_ [22].

It was also concluded that TiO_2_–SnO_2_ (90:10) wt.% nanocomposite has the lowest band gap value (2.8 eV), so (90:10) wt.% is the optimum ratio, which means it needs lower energy for excitation [23] as shown in Table 2.

**Table 2:** Effect of SnO_2_ percentage on TiO_2_–SnO_2_ nanocomposite band gap energy

Sample	Band gap energy (eV)
TiO_2_	3.2
SnO_2_	3.6
TiO_2_–SnO_2_ (97:3) wt.%	2.91
TiO_2_–SnO_2_ (93:7) wt.%	2.88
**TiO**_**2**_–**SnO**_**2**_** (90:10) wt.%**	**2.80**
TiO_2_–SnO_2_ (85:15) wt.%	2.82
TiO_2_–SnO_2_ (80:20) wt.%	2.86

### Photocatalysis of acid red 37 dye

The photocatalytic degradation of aqueous acid red 37 dye solution was studied with different AOPs using UV/TiO_2_, UV/SnO_2_, UV/TiO_2_–SnO_2_ (97:3) wt.%, UV/TiO_2_–SnO_2_ (93:7) wt.%, UV/TiO_2_–SnO_2_ (90:10) wt.%, UV/TiO_2_–SnO_2_ (85:15) wt.%, UV/TiO_2_–SnO_2_ (80:20) wt.%, UV/TiO_2_–SnO_2_ (90:10)/H_2_O_2_, UV/TiO_2_–SnO_2_ (90:10)/S_2_O_8_ and UV/TiO_2_–SnO_2_ (90:10)/IO_4_^−^. Then, the effect of pH, dye concentrations, and different loading (g/L) of nanocomposite on the photocatalytic activity of the TiO_2_–SnO_2_ nanocrystals were investigated in every experiment, the batch photoreactor was employed as the photoreactor.

### Effect of native TiO_2_, SnO_2_ and TiO_2_–SnO_2_ nanocomposites as photocatalysts

**Fig. 12:**
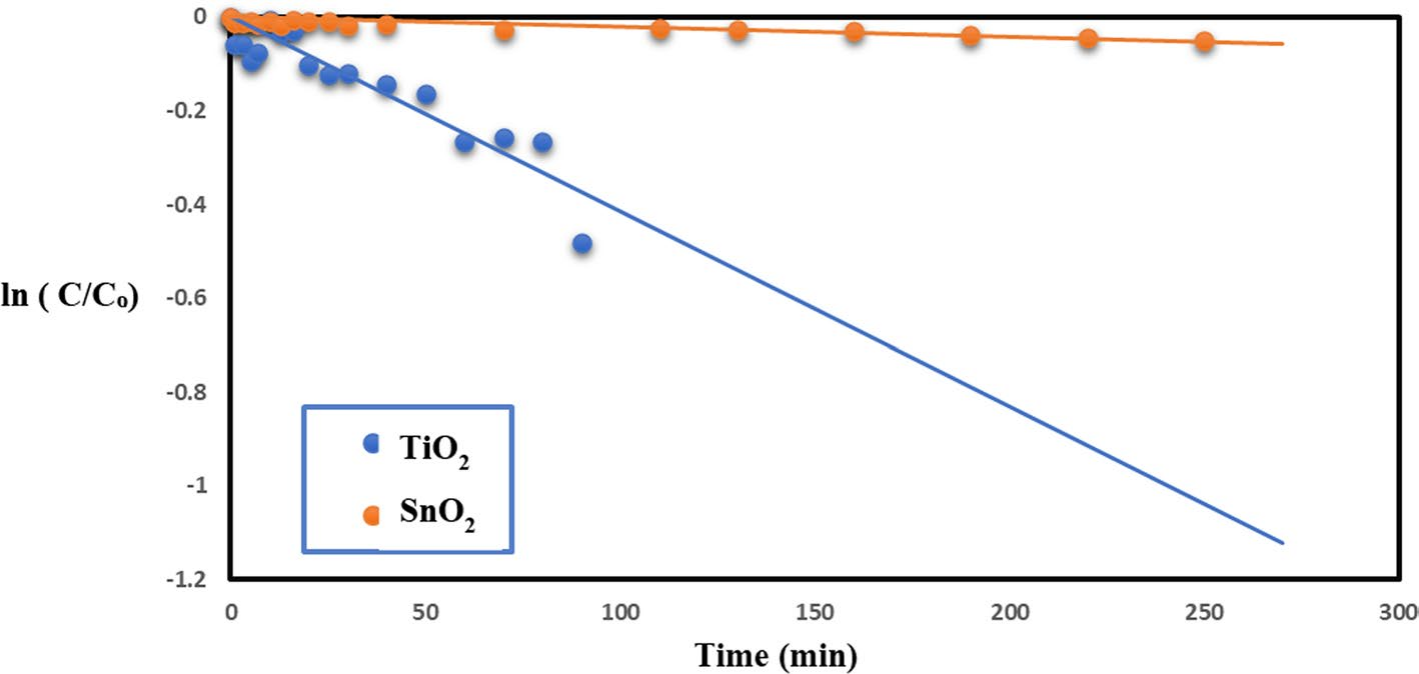
Change of ln (C/C_o_) with time for photocatalytic degradation of acid red 37 dye using 0.4 g/L of TiO_2_ or SnO_2_

Photodegradation occurred when UV light at a wavelength of 254 nm irradiated slurry solutions of TiO_2_ and SnO_2_ 0.4 (g/L) containing acid red 37 dye, as illustrated in Fig. 12. This is illustrated in Fig. 1, as stated in the introduction [18–21]. A pseudo first-order model for dye degradation was employed to illustrate the data. The slopes of the linear graphs depicting.

ln (C/Co) with time, where Co denotes the initial concentration of acid red 37 dye and C signifies the dye concentration at time t, were utilized to compute the values of the first-order rate constant (k_app_). The duration required for the reactants to be reduced to half of their initial concentrations is referred to as the half-life (t_1/2_) of a first-order reaction. The half-life (t_1/2_) and the rate constant (k_app_) are inversely related, as demonstrated by Eq. (2):

2 $$ {\text{t}}_{{{1}/{2}}} = \, 0.{693 }/{\text{ k}}_{{{\text{app}}}} $$

It was discovered that increasing the apparent rate constant (k_app_) causes the t_1/2_ to decrease, as indicated in Table 3.

**Table 3:** The apparent rate constants and half-life times for photocatalytic degradation of acid red 37 dye using UV/TiO_2_, UV/SnO_2_, UV/TiO_2_–SnO_2_ (97:3) wt.%, UV/TiO_2_–SnO_2_ (93:7) wt.%, UV/TiO_2_–SnO_2_ (90:10) wt.%, UV/TiO_2_–SnO_2_ (85:15) wt.%, and UV/TiO_2_–SnO_2_ (80:20) wt.%

Catalytic system	Catalyst Loading (g/L)	k_app_ (min^−1^)	t_1/2_ (min)
UV/TiO_2_	0.4	2 × 10^–4^	3460
UV/SnO_2_	0.4	28 × 10^–4^	247.1
UV/TiO_2_–SnO_2_ (97:3) wt.%	0.4	38 × 10^–4^	182.1
UV/TiO_2_–SnO_2_ (93:7) wt.%	0.4	44 × 10^–4^	157.2
UV/TiO_2_–SnO_2_ (90:10) wt.%	0.4	72 × 10^–4^	96.1
UV/TiO_2_–SnO_2_ (85:15) wt.%	0.4	47 × 10^–4^	147.2
UV/TiO_2_–SnO_2_ (80:20) wt.%	0.4	35 × 10^–4^	197.7

Irradiation of SnO_2_ 0.4 (g/L) with UV light of wavelength 254 nm resulted in a lower photodegradation of acid red 37 dye compared with UV/TiO_2_. But SnO_2_ exhibited good catalytic performance and stability. The superior photocatalytic activity of TiO_2_ over SnO_2_ may be attributed to smaller TiO_2_ particle size, quick (e^−^/h^+^) pair recombination rate, and poor quantum yield of SnO_2_ in aqueous solutions [24].

**Fig. 13:**
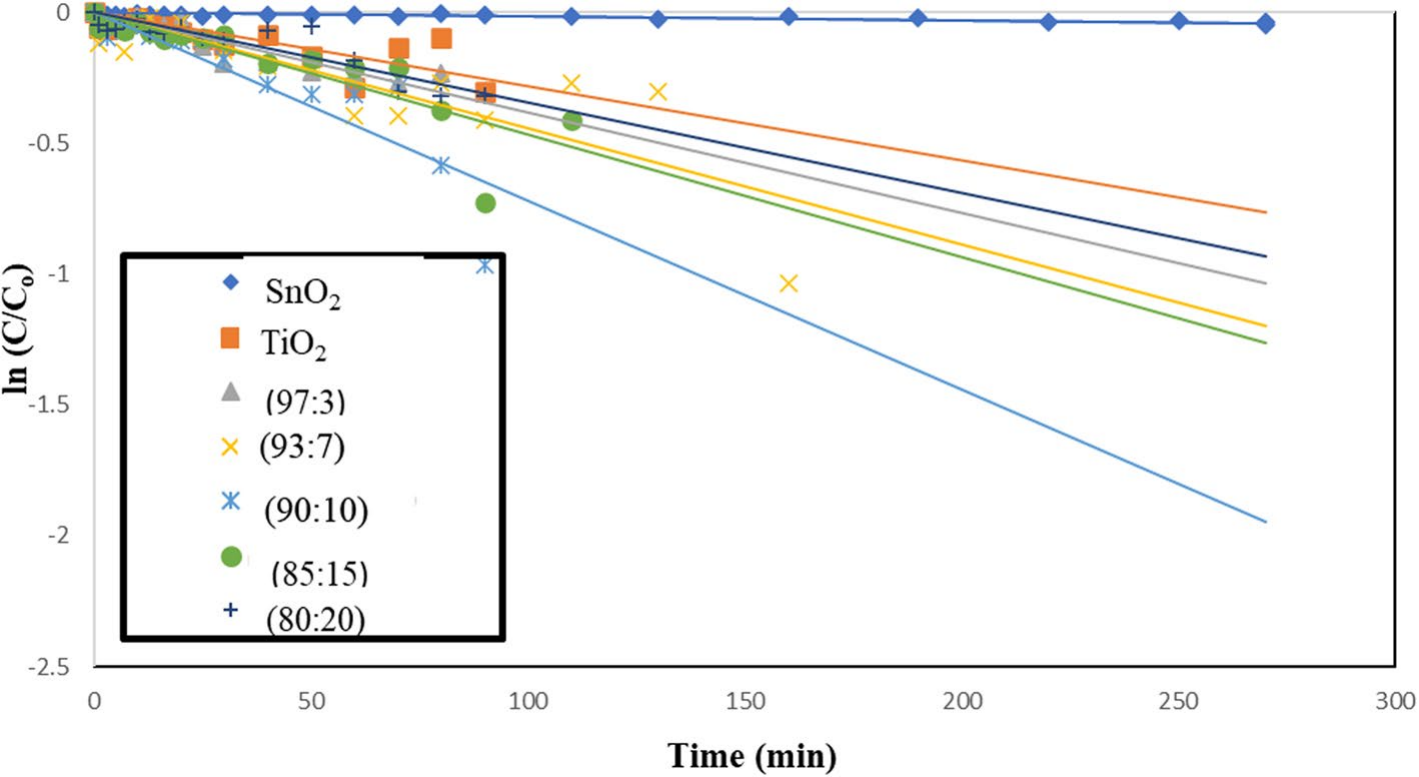
Change of ln (C/C_o_) with time for photocatalytic degradation of acid red 37 dye using UV/TiO_2_, UV/SnO_2_, UV/TiO_2_–SnO_2_ (97:3) wt.%, UV/TiO_2_–SnO_2_ (93:7) wt.%, UV/TiO_2_–SnO_2_ (90:10) wt.%, UV/TiO_2_–SnO_2_ (85:15) wt.% and UV/TiO_2_ SnO_2_(80:20) wt.%

**Fig. 14:**
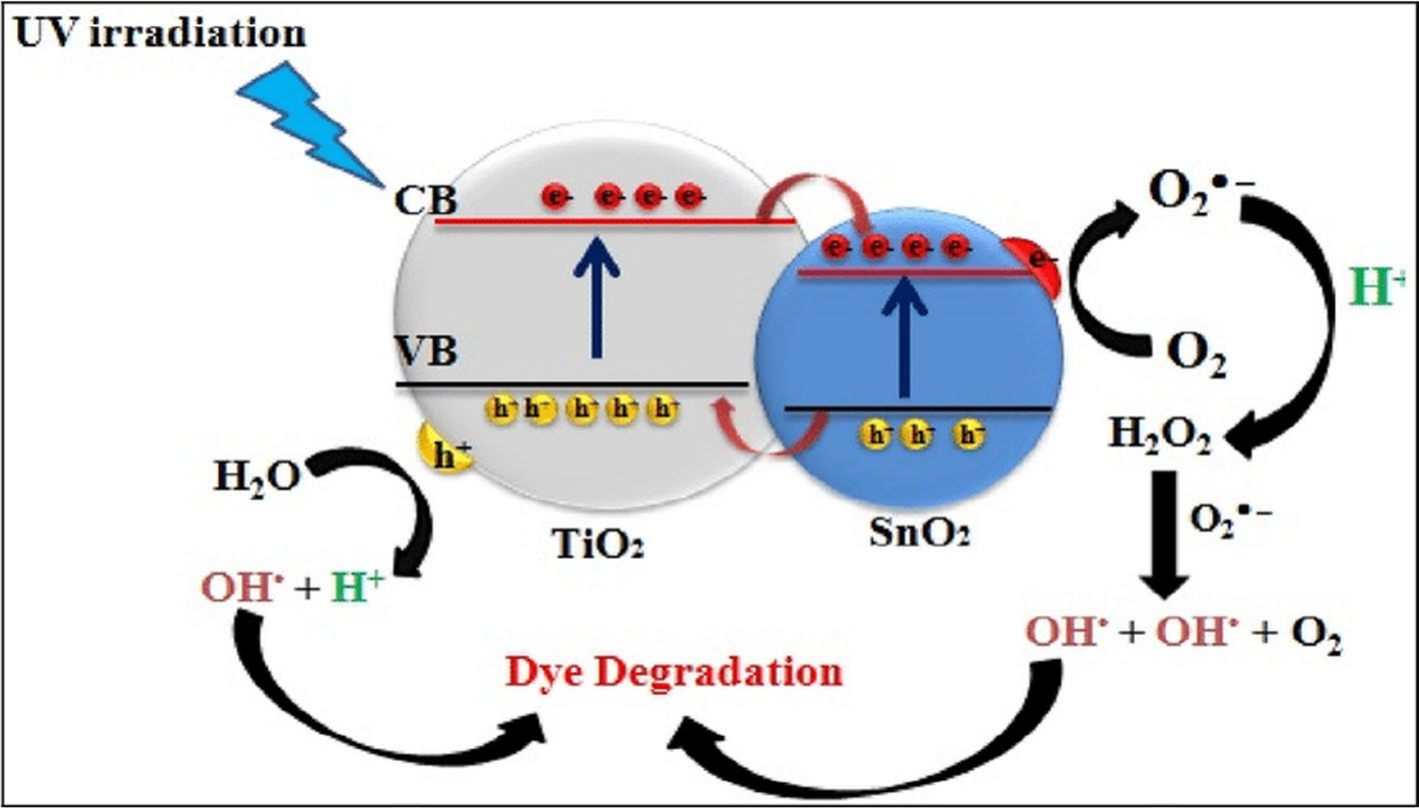
Relative energy position of the bands and the process of charge separation in the TiO_2_–SnO_2_ system

Figure 13 indicates that the produced TiO_2_–SnO_2_ nanocomposite demonstrates superiority to photocatalytic activity relative to natural TiO_2_ or SnO_2_. The elevated photocatalytic activity of the TiO_2_–SnO_2_ composite is ascribed to the enhanced separation of photoinduced electron–hole (e^−^/h^+^) pairs; notably, the n-p heterojunction of TiO_2_–SnO_2_ facilitates the migration of photogenerated holes towards the interface and electrons towards the bulk. Due to the conduction band of SnO_2_ for being more positive than that of TiO_2_ to stimulate electrons in TiO_2_, can migrate to SnO_2_, while photogenerated holes can be a transition to the valence band of SnO_2_ to result in the separation of (e^−^/h^+^) pairs. This is illustrated in Fig. 14. Compared to native TiO_2_ or SnO_2_, the TiO_2_–SnO_2_ nanocomposite exhibited significantly enhanced photocatalytic activity. The degradation of acid red 37 dye through photocatalysis is greatly influenced by the concentration of SnO_2_ in the TiO_2_–SnO_2_ nanocomposite. It was found that augmentation of the SnO_2_ concentration from (3 to 10) wt.% led to an enhanced photocatalytic degradation of the dye. This may result from a reduction in the band gap, as indicated by band gap calculations for the TiO_2_–SnO_2_ (90:10) wt.% nanocomposite, which exhibited the lowest band gap to prevent recombination and diminish the time required for electron and hole transfer between the conduction band and the valence band, thereby, enhancing photocatalytic activity. The reduction in photocatalytic degradation of the dye with escalating SnO_2_ content from (15 to 20) wt.% may result from the formation of SnO_2_–SnO_2_ homojunctions which diminish the quantity of TiO_2_–SnO_2_ heterojunctions and concurrently augment the number of free SnO_2_ particles, characterized by low photocatalytic activity [18].

### Effect of addition of various inorganic oxidants to TiO_2_–SnO_2_ (90:10) nanocomposite

#### Effect of addition of H_2_O_2_ to TiO_2_–SnO_2_ (90:10) wt.% nanocomposite

**Fig. 15:**
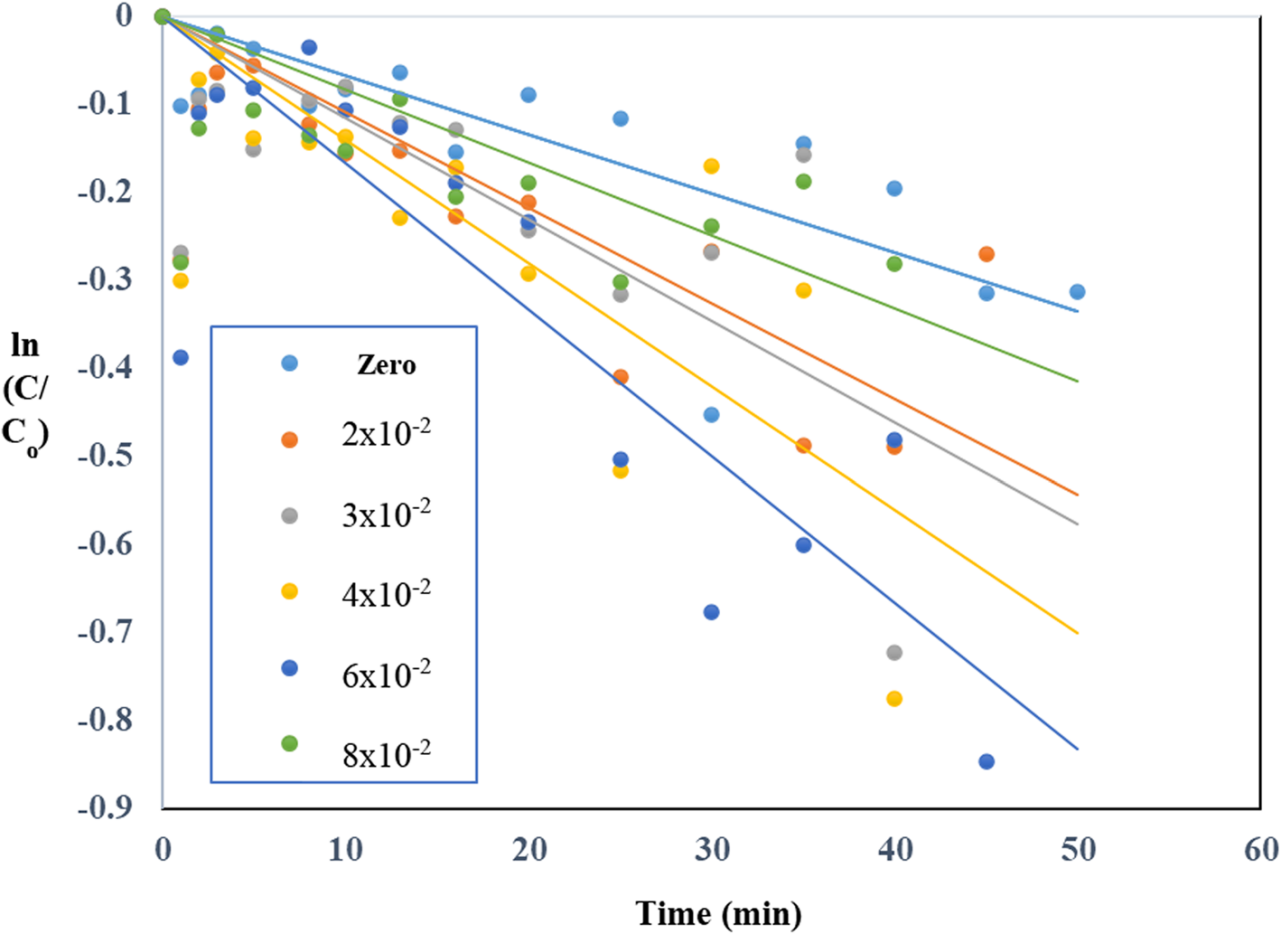
Change of ln (C/C_o_) with time for photocatalytic degradation of acid red 37 dye using 0.4 g/L of TiO_2_–SnO_2_ (90: 10) wt.% with different concentrations of H_2_O_2_ (M)

TiO_2_–SnO_2_ (90: 10) wt.% nanocomposite with SnO_2_ (10 wt.%) was utilized to explore the influence of several oxidants (H_2_O_2_, Na_2_S_2_O_8_ and NaIO_4_) on the photocatalytic breakdown of acid red 37 dye based on earlier findings. Figure 15 shows the effect of adding different concentrations of hydrogen peroxide to TiO_2_–SnO_2_ (90:10) 0.4 (g/L) for photodegradation of acid red 37 dye under UV irradiation [25]. The data was plotted using a first-order dye destruction model. Adding low concentration of hydrogen peroxide (2 × 10^–2^ M) to UV/TiO_2_–SnO_2_ (90:10) wt.% 0.4 (g/L) increases the rate of photocatalytic degradation of acid red 37 dye when compared with UV/TiO_2_–SnO_2_ (90:10) wt.% alone. The rate of photodegradation increased when the concentration of hydrogen peroxide was increased to 6 × 10^–2^ M with k_app_ ranged from 789 X10^−4^ to 997 X10^−4^ min^−1^, then decreased with further increasing of hydrogen peroxide concentration to 8 × 10^–2^ M with k_app_ of 698 × 10^–2^ M (Table 4).

**Table 4:** The apparent rate constants, half-life times, and reaction order for photocatalytic degradation of acid red 37 dye using UV/TiO_2_–SnO_2_ (90:10) wt.%/H_2_O_2_

Catalytic system	Concentration of H_2_O_2_ (M)	k_app_ (min^−1^)	t_1/2_ (min)	Apparent reaction order (n)
UV/TiO_2_–SnO_2_ (90:10) wt.% (0.4 g/L)/H_2_O_2_	2 × 10^–2^	789 × 10^–4^	8.7	2.3
	3 × 10^–2^	883 × 10^–4^	7.8	
	4 × 10^–2^	943 × 10^–4^	7.3	
	6 × 10^–2^	997 × 10^–4^	6.9	
	8 × 10^–2^	698 × 10^–4^	9.9	

**Fig. 16:**
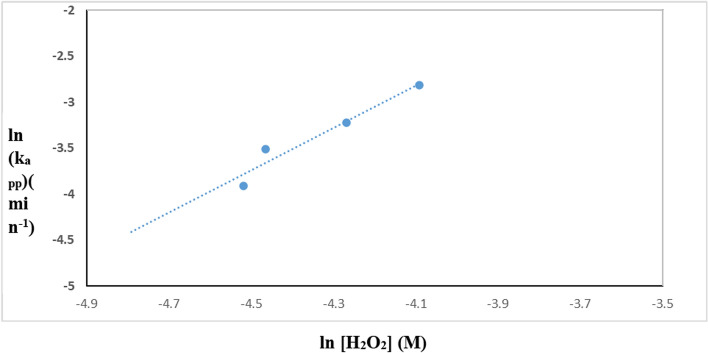
Change of ln k_app_ (min^−1^) with ln [H_2_O_2_] (M) for photocatalytic degradation of acid red 37 dye

A power law relationship describes the effect of rate-determining species Eq. (3):

3 $$ {\text{k}}_{{{\text{app}}}} = {\text{ K}} \left[ {{\text{H}}_{{2}} {\text{O}}_{{2}} } \right]^{{\text{n}}} $$

where k _app_ and K are the apparent and true rate constants, respectively. Plotting the logarithm of k_app_ against the logarithm of the concentration of hydrogen peroxide in Fig. 16 produced a value of 2.3 for the exponent, n, the order of reaction with regard to the oxidant species (Table 4). According to the following reaction, the photogenerated conduction band electrons of TiO_2_–SnO_2_ are more efficiently trapped by H_2_O_2_ than they are by O_2_, which may explain why the rate of dye degradation is increased when using UV/TiO_2_–SnO_2_ (90: 10) wt.%/H_2_O_2_ system as compared with UV/TiO_2_–SnO_2_ (90:10) wt.% alone in Eq. (4) [26, 27]:

4 $$ {\text{H}}_{2} {\text{O}}_{2} + {\text{ e}}^{ - }_{(cb)} \to^{ - } {\text{OH }} +^{ \cdot } {\text{OH}} $$

Additional oxidizing species (^·^OH), can generally aid in the oxidative decay process. Besides, the scavenging action slow the recombination interaction between photogenerated carriers, (electrons and holes). Since the employed UV source's main output was at 254 nm, significant photolysis of the H_2_O_2_ would have taken place, creating additional hydroxyl radicals [28, 29].

5 $$ {\text{H}}_{2} {\text{O}}_{2} + \, h\nu \, \to \, 2^{ \cdot } {\text{OH}} $$

By raising the H_2_O_2_ concentrations (2 × 10^–2^–6 × 10^–2^) M, dye oxidation and degradation are accelerated as well as the electron scavenging. Further increasing in H_2_O_2_ concentration to × 10^–2^ M decreases the photocatalytic degradation rate due to the recombination of the resulting of (OH) radicals which occurs at high concentration of H_2_O_2_.

### Effect of addition of S_2_O_8_^2−^ to TiO_2_–SnO_2_ (90:10) wt.% nanocomposite

The oxygen on the surface of irradiated TiO_2_–SnO_2_ (90: 10) wt.% suspension acts as a natural sink for the photogenerated conduction band electrons. The sorbed H_2_O or hydroxyl ions on TiO_2_–SnO_2_ surface are subsequently oxidized by the remaining holes to produce the OH radicals.

It could be advantageous to use an electron acceptor other than oxygen that is more effective [30–32].

**Fig. 17:**
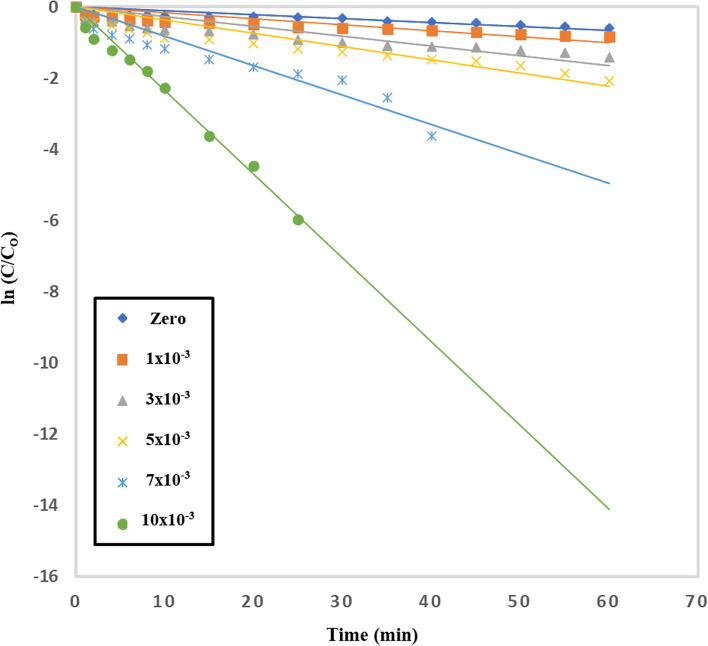
Change of ln (C/C_o_) with time for photocatalytic degradation of acid red 37 dye using 0.4 (g/L) of TiO_2_–SnO_2_ (90: 10) wt.% with different concentrations of S_2_O_8_^2−^ (M)

**Fig. 18:**
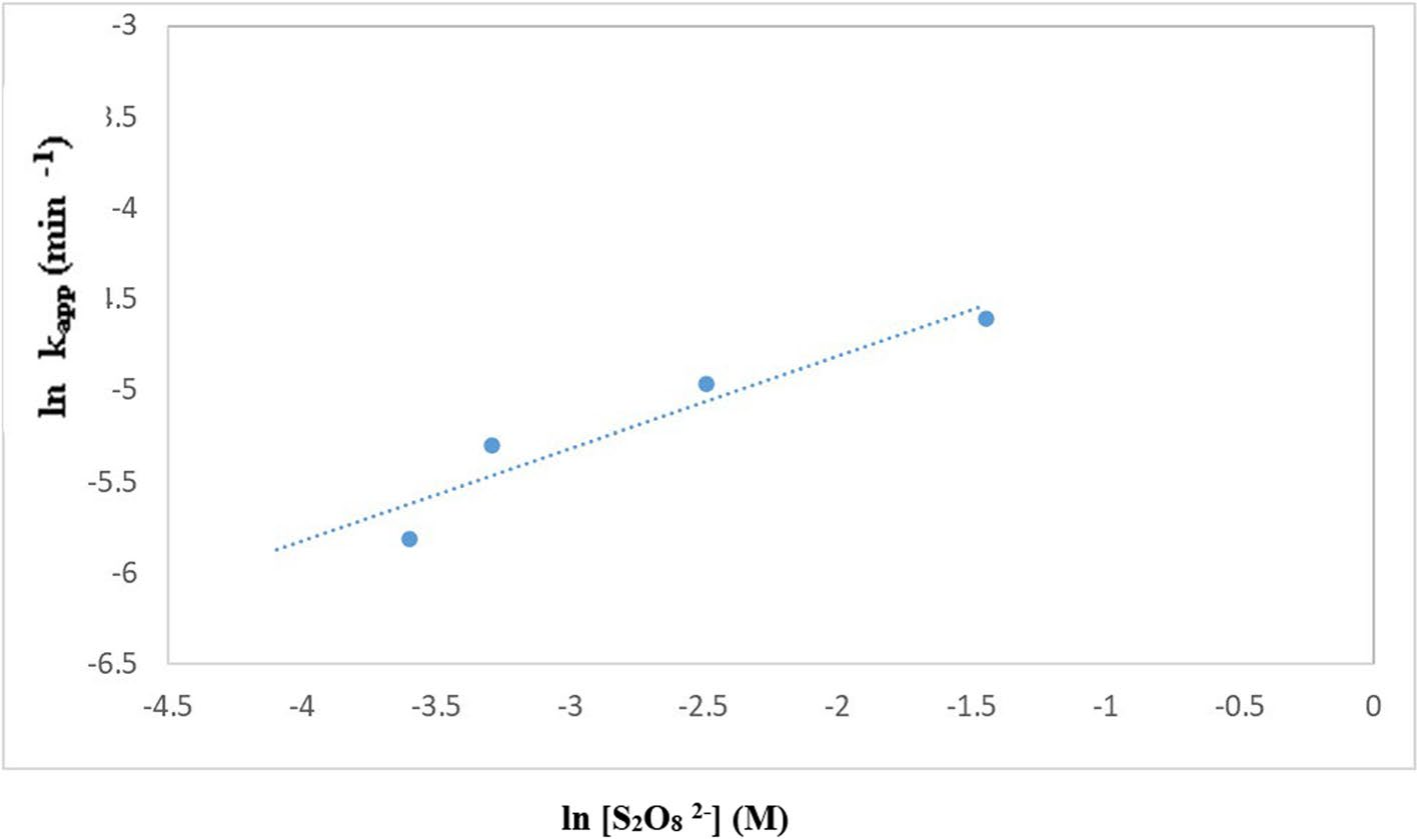
Change of ln k_app_ (min^−1^) with ln [S_2_O_8_^2−^] (M) for photocatalytic degradation of acid red 37 dye

The impact of adding persulfate to TiO_2_–SnO_2_ (90:10) wt.% 0.4 (g/L) on the photocatalytic degradation of acid red 37 dye is shown in Fig. 17. The rate of deterioration using TiO_2_–SnO_2_ (90:10) 0.4 (g/L) was found to be accelerated by adding a little amount of persulfate (1.0 × 10^–3^ M). The dye had degraded completely and quickly when the persulfate concentration was increasing to 10 × 10^–3^ M (2.9 min). Figure 18 shows the reaction rate order regarding persulfate, and it was determined to be 0.5 (Table 5). The persulfate anions may capture the photogenerated conduction band electrons of TiO_2_–SnO_2_ more than O_2_ or H_2_O_2_ and produce the powerful oxidizer SO_4_^·−^ [31].

6 $$ {\text{S}}_{2} {\text{O}}_{8}^{2 - } + {\text{ e}}^{ - }_{{({\text{cb}})}} \to {\text{ SO}}_{4}^{2 - } + {\text{ SO}}_{4}^{ \cdot - } $$

**Table 5:** The apparent rate constants, half-life times, and reaction order for photodegradation of acid red 37 dye using UV/TiO_2_–SnO_2_ (90:10)/S_2_O_8_^2−^

Catalytic system	Concentration of [S_2_O_8_^2−^] (M)	k_app_ (min^−1^)	t_1/2_ (min)	Apparent reaction order (n)
UV/TiO_2_–SnO_2_ (90:10) wt.% 0.4 (g/L)/S_2_O_8_^2−^	1 × 10^–3^	166 × 10^–4^	41.7	0.5
	3 × 10^–3^	273 × 10^–4^	25.3	
	5 × 10^–3^	371 × 10^–4^	18.6	
	7 × 10^–3^	826 × 10^–4^	8.3	
	10 × 10^–3^	2352 × 10^–4^	2.9	

Furthermore, at a wavelength of 254 nm, the sulphate radical anion can interact with the solvent and produce ^·^OH in the following processes [33–35]:

7 $$ {\text{S}}_{2} {\text{O}}_{8}^{2 - } + \, h\nu \, \to \, 2{\text{ SO}}_{4}^{ \cdot - } $$

8 $$ {\text{SO}}_{4}^{ \cdot - } + {\text{ H}}_{2} {\text{O }} \to^{ \cdot } {\text{OH }} + {\text{ SO}}_{4}^{2 - } + {\text{ H}}^{ + } $$

As a result, increasing the persulfate concentrations (1 × 10^–3^–10 × 10^–3^) M improved both the trapping of photogenerated conduction band electrons and the production of SO_4_^·−^and ^·^OH which led to a higher rate of photocatalytic degradation.

### Effect of addition of IO_4_^−^ to TiO_2_–SnO_2_ (90:10) wt.% nanocomposite

**Fig. 19:**
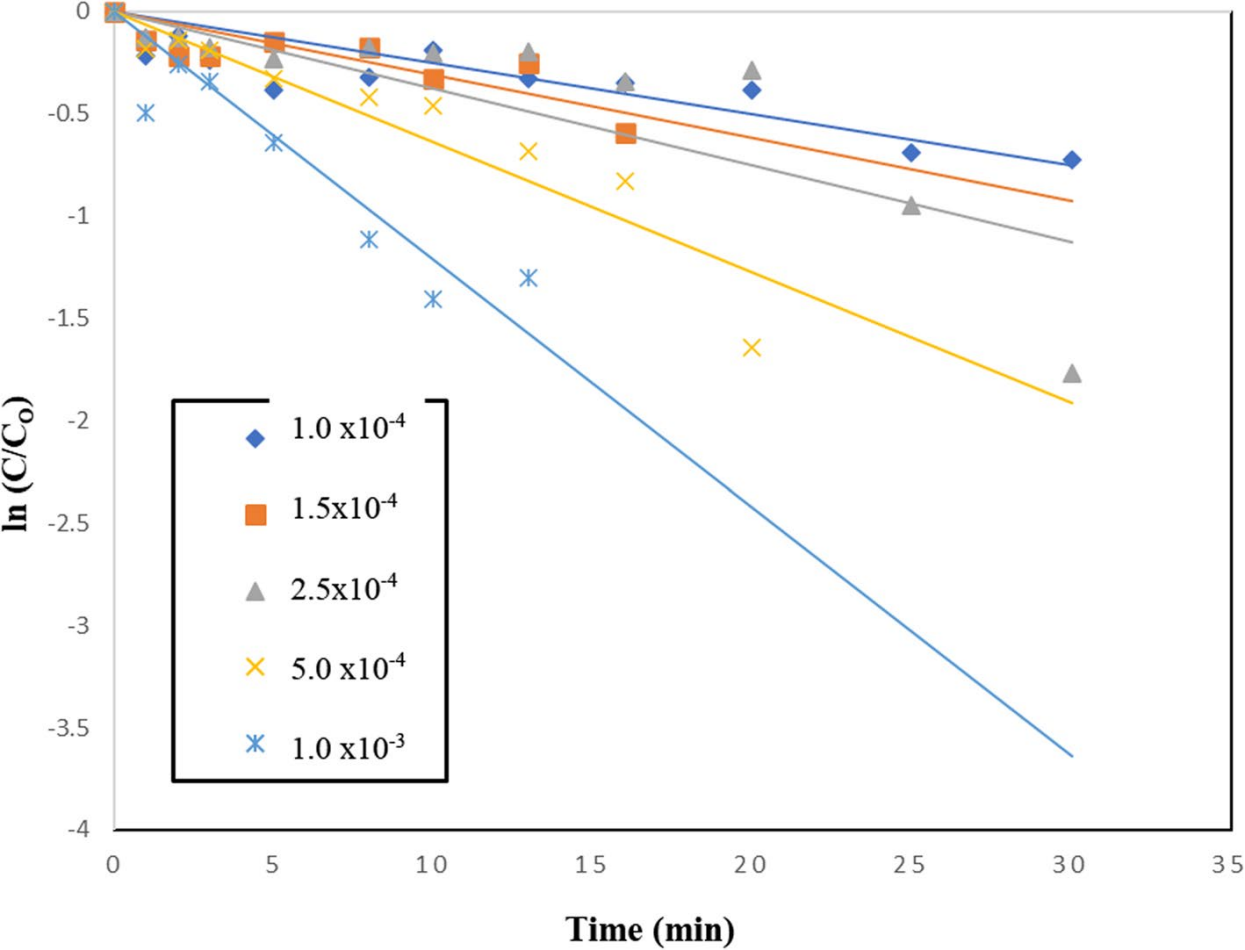
Change of ln (C/C_o_) with time for photocatalytic degradation of acid red 37 dye using 0.4 (g/L) of TiO_2_–SnO_2_ (90: 10) wt.% with different concentrations of IO_4_^−^ (M)

The effect of adding periodate to TiO_2_–SnO_2_ (90:10) wt.% 0.4 (g/L) for photocatalytic degradation of the studied acid red 37 dye is shown in Fig. 19. It can be seen that the addition of very low concentration of periodate (1 × 10^−4^ M) to UV/TiO_2_–SnO_2_ (90:10) wt.% 0.4 (g/L) resulted in higher photocatalytic degradation rate compared with UV/TiO_2_–SnO_2_ (90–10) wt.% 0.4 (g/L) system alone. Also, increasing the periodate concentration to 1 × 10^–3^ resulted in higher photocatalytic degradation of the dye compared with UV/TiO_2_–SnO_2_ (90:10) wt.% 0.4 (g/L)/H_2_O_2_ or UV/TiO_2_–SnO_2_ (90:10) wt.% 0.4 (g/L)/S_2_O_8_^2−^ systems, indicating the effectiveness of periodate over peroxide or persulfate.

**Fig. 20:**
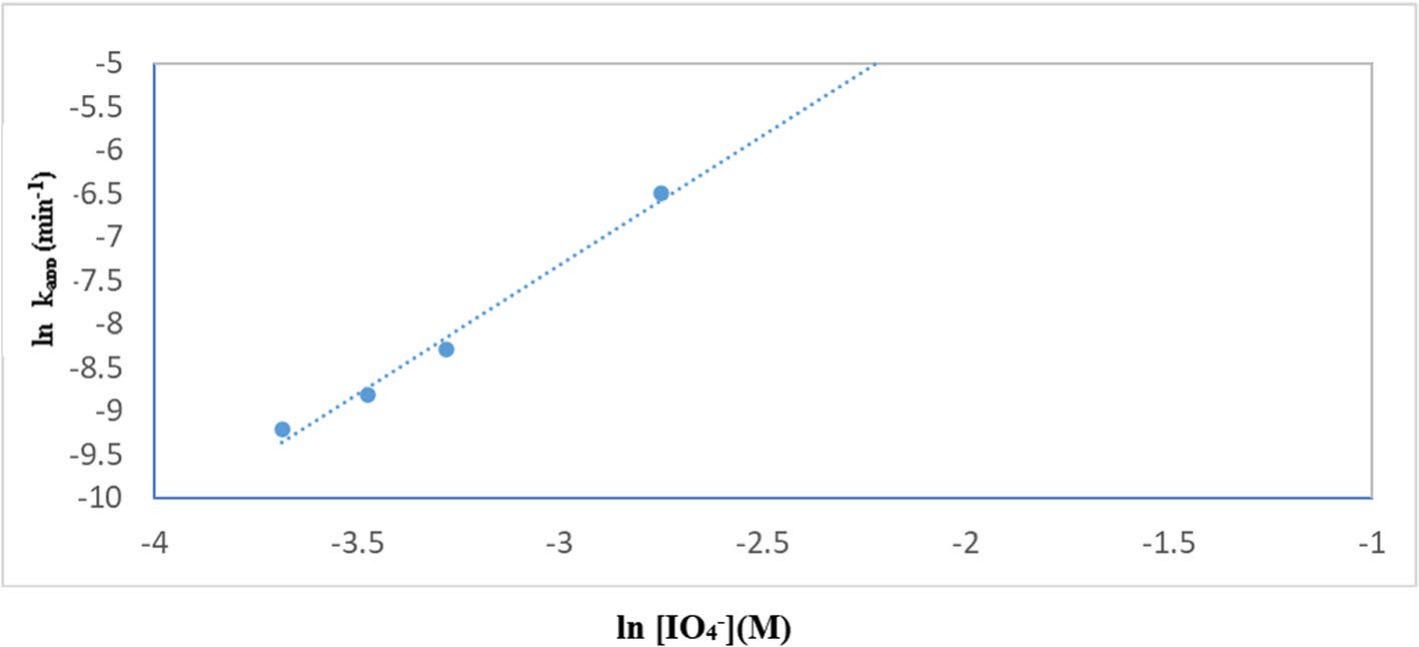
Change of ln k_app_ (min^−1^) with ln [IO_4_^−^] (M) for photocatalytic degradation of acid red 37 dye

The reaction rate order with respect to periodate was found to be 2.9 (Fig. 20 and Table 6). The enhancement of the dye degradation may be due to the scavenging of the photogenerated conduction band electrons of the excited TiO_2_–SnO_2_ by periodate which is more efficient than trapping with O_2_, H_2_O_2_ or S_2_O_8_^2−^ as follows [36]:

9 $$ {\text{IO}}_{4}^{ - } + 8{\text{e}}^{ - }_{{({\text{cb}})}} + \, 8{\text{H}}^{ + } \to \, 4{\text{H}}_{2} {\text{O }} + {\text{I}}^{ - } $$

**Table 6:** The apparent rate constants, half-life times, and reaction order for photodegradation of acid red 37 dye using UV/TiO_2_–SnO_2_ (90–10)/IO_**4**_^**−**^

Catalytic system	Concentration of IO_4_^−^ (M)	k_app_ (min^−1^)	t_1/2_ (min)	Apparent reaction order (n)
UV/TiO_2_–SnO_2_ (90:10) wt.% 0.4 (g/L)/IO_4_^−^	1.0 × 10^–4^	251 × 10^–4^	27.6	2.9
	1.5 × 10^–4^	31 × 10^–4^	22.3	
	2.5 × 10^–4^	376 × 10^–4^	18.4	
	5.0 × 10^–4^	638 × 10^–4^	10.8	
	1.0 × 10^–3^	1214 × 10^–4^	5.7	

In addition, under UV irradiation (254 nm), a variety of highly reactive radical and non-radical intermediates (IO_3_^·^, ^·^OH, and IO_4_^·^) are produced during the photolytic degradation of periodate which enhanced the degradation rate [37]:

10 $$ {\text{IO}}_{4}^{ - } + \, h\nu \, \to {\text{ IO}}_{3}^{ \cdot } + {\text{ O}}^{ \cdot - } $$

11 $$ {\text{O}}^{ \cdot - } + {\text{ H}}^{ + } \leftrightarrow^{ \cdot } {\text{OH}} $$

12 $$^{ \cdot } {\text{OH }} + {\text{ IO}}_{4}^{ - } \to {\text{ OH}}^{ - } + {\text{IO}}_{4}^{ \cdot } $$

An increase in periodate concentration enhances the generation of IO_3_^·^, O^·^, ^·^OH, and IO_4_^·^, as well as the trapping of photogenerated conduction band electrons of TiO_2_ and SnO_2_ (90:10).

Qamar et al. [38] had shown that the degradation of chrysoidine R and Acid Red 29 dyes was hastened when peroxide or periodate was added to UV light containing TiO_2_. It was discovered that the response rate followed the sequence:

$$ {\text{UV}}/{\text{TiO}}_{{2}} /{\text{S}}_{{2}} {\text{O}}_{{8}}^{{{2} - }} > {\text{ UV}}/{\text{TiO}}_{{2}} /{\text{H}}_{{2}} {\text{O}}_{{2}} > {\text{ UV}}/{\text{TiO}}_{{2}} $$

Vučić et al. [39] found that H_2_O_2_, KBrO_3_, and (NH_4_)2S_2_O_8_ led to increase the photocatalytic degradation of the chromotrope anthraquinone dye Reactive Blue 19. The rate of degradation was around:

$$ {\text{UV}}/{\text{TiO}}_{{2}} /{\text{BrO}}_{{3}}^{ - } > {\text{ UV}}/{\text{TiO}}_{{2}} /{\text{S}}_{{2}} {\text{O}}_{{8}}^{{{2} - }} > {\text{ UV}}/{\text{TiO}}_{{2}} /{\text{H}}_{{2}} {\text{O}}_{{2}} > {\text{ UV}}/{\text{TiO}}_{{2}} $$

### Effect of pH

**Fig. 21:**
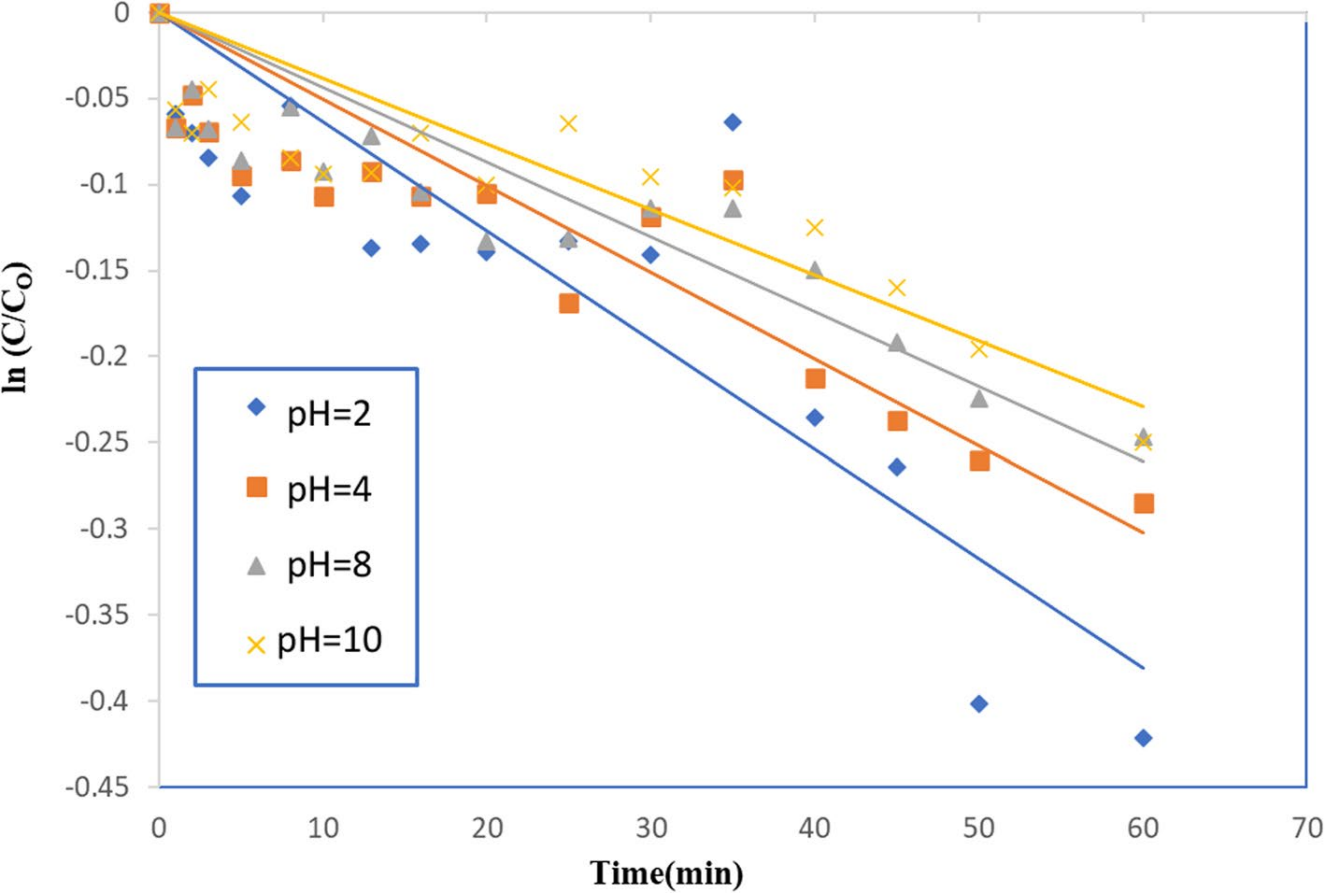
Photocatalytic degradation of acid red 37 (1.0 × 10^–4^ M), using 0.4 (g/L) TiO_2_–SnO_2_ nanocomposite at different pH values

Figure 21 shows how the pH of the solution affects the photocatalytic breakdown of the acid red dye 37 using 0.4 g/L (90:10) TiO_2_–SnO_2_ wt.%. At the same dye concentration and the catalyst amount within the same temperature, the increasing of the pH from 2 to 10 results in a decrease in photocatalytic activity. At acidic pH's, if sulfonate groups are present and the dye is negatively charged (SO_3_^−^ Na^+^) and the catalyst surface is positively charged, a faster degradation rate was observed (at acidic pH). Accelerated photocatalytic degradation is caused by the electrostatic interaction of the dye molecules with the catalyst surface [40]. The photocatalytic degradation of acid red 37 dye k_app_ (min^−1^) using 0.4 (g/L) TiO_2_–SnO_2_ at different pH’s. The reaction rate order was found to be 3.3 (Table 7). It is determined that increasing the pH of the solution causes the k_app_ (min^−1^) level to decrease and the t_1/2_ (min) to increase.

**Table 7:** The apparent rate constant and half lifetime for photocatalytic degradation of acid red 37 dye using 0.4 (g/L) TiO_2_–SnO_2_ nanocomposite at different pH values

Catalytic system	PH	k_app_ (min^−1^)	t_1/2_ (min)	Apparent reaction order (n)
UV/TiO_2_–SnO_2_ (90:10) 0.4(g/L)	2	70 × 10^–4^	99	3.3
	4	63 × 10^–4^	110	
	8	44 × 10^–4^	157.5	
	10	38 × 10^–4^	182.4	

### Effect of acid red 37 concentration

**Fig. 22:**
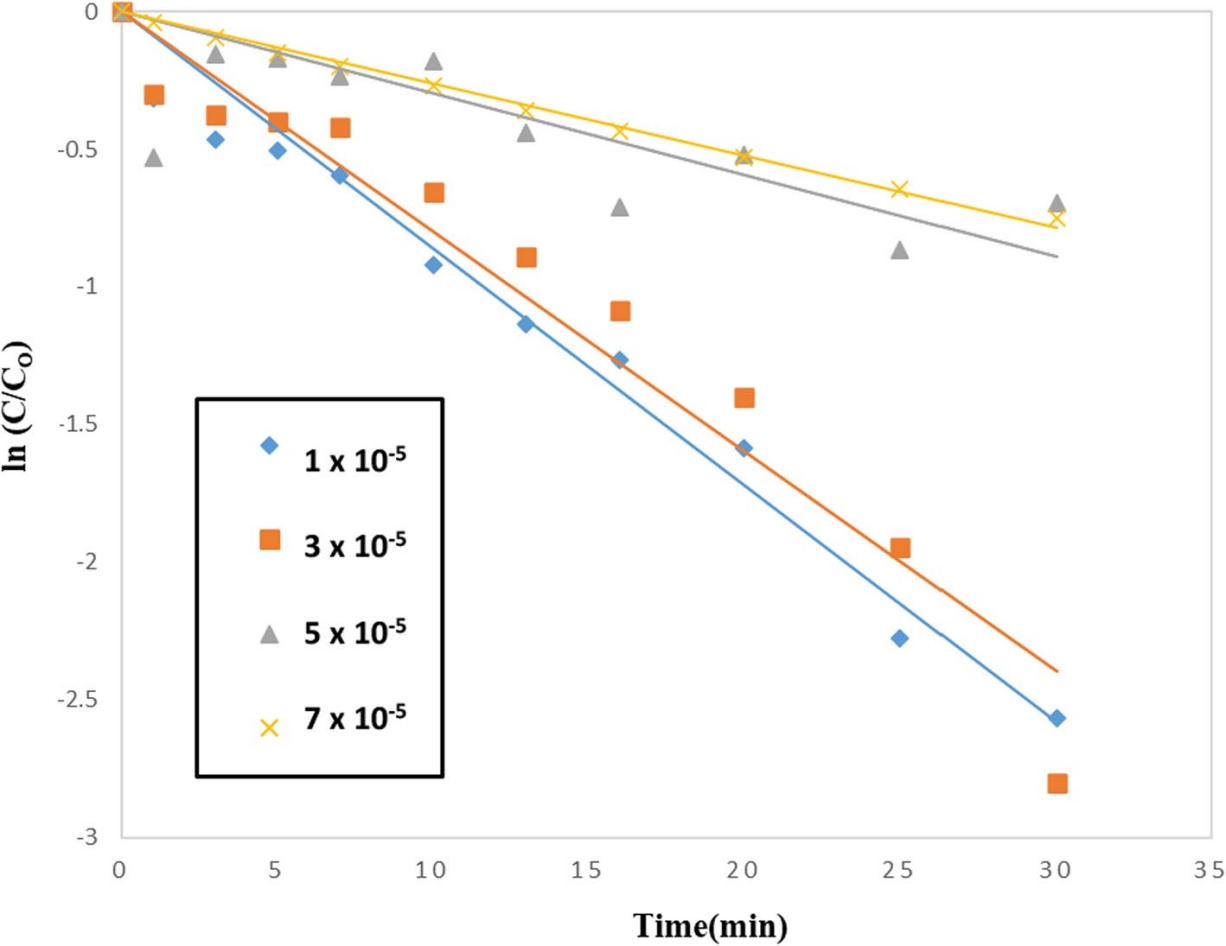
Photocatalytic degradation of acid red 37 (1.0 × 10^–4^ M), using 0.4 (g/L) TiO_2_–SnO_2_ nanocomposite at different dye concentration

**Fig. 23:**
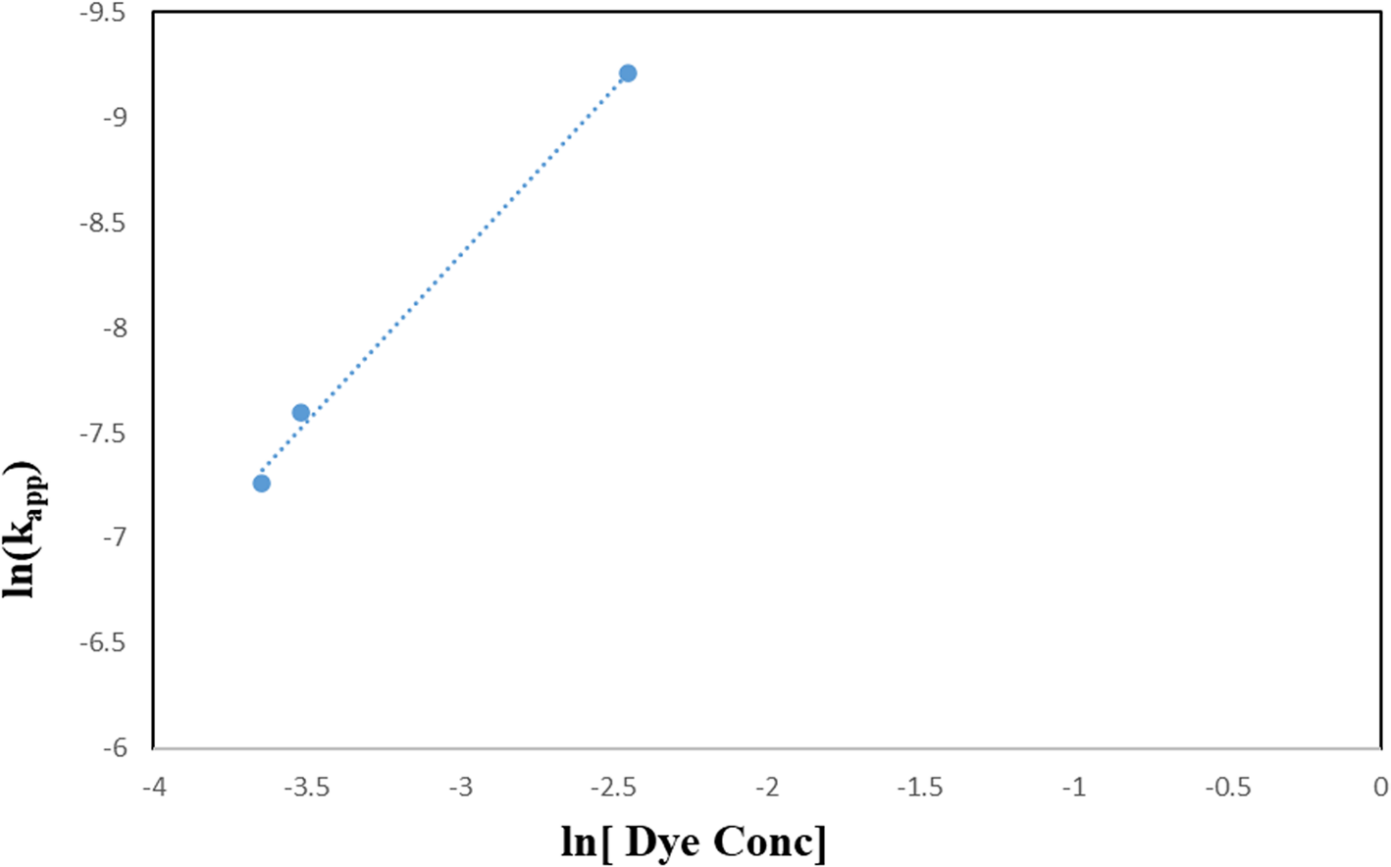
Plot of ln k_app_ (min^−1^) versus ln [Dye Conc] for photocatalytic degradation of acid red 37 dye at different dye concentration

In general, the starting dye concentration in each photocatalytic reaction is an important parameter that must be considered. The photocatalytic process is dependent on dye adsorption on the photocatalyst surface. As a result, the amount of dye that can be used in a photocatalytic reaction is only as much as what can be adsorbed on the surface of the photocatalyst. Furthermore, as the dye concentration increases when the quantity of catalyst is constant, the rate of photocatalytic degradation declines. This may be explained by the fact that when the dye concentration increases, more organic molecules are adsorbed on the TiO_2_–SnO_2_ surface while less photons reach the catalyst surface, resulting in fewer hydroxyl radicals being formed with less degradation [41]. Figure 22 illustrates the photocatalytic degradation of different concentrations of acid red 37 dye under UV illumination and 0.4 (g/L) photocatalyst TiO_2_–SnO_2_ conditions. The apparent rate constant and half-life time for the photocatalytic degradation of acid red 37 dye are also shown in Fig. 23 for different dye concentrations Table 8. The results revealed that the k_app_ (min^−1^) values decreased while the t_1/2_ (min^−1^) values increased as the dye concentration increased.

**Table 8:** The apparent rate constant and half-life time for photocatalytic degradation of different concentrations of acid red 37 dye under UV irradiation acid red 37 dye using 0.4 (g/L) TiO_2_–SnO_2_ nanocomposite

Catalytic system	Concentration of the dye (M)	k_app_ (min^−1^)	t_1/2_ (min)	Apparent reaction order (n)
UV/TiO_2_–SnO_2_ (90:10) 0.4(g/L)	0.01 × 10^–3^	861 × 10^–4^	8	1.5
	0.03 × 10^–3^	799 × 10^–4^	8.6	
	0.05 × 10^–3^	297 × 10^–4^	23	
	0.07 × 10^–3^	261 × 10^–4^	26	

### Effect of using TiO_2_–SnO_2_ (90–10) wt.% nanocomposite at different loading (g/L)

Under UV-light (= 254 nm), the acid red 37 dye was photocatalytically degraded and 1.0 × 10^–4^ M of the dye, both with and without the prepared photocatalyst, was observed in the following manner where absence of the prepared nano catalyst, no degradation of the dye occurs, and the stability of the dye. However, upon using different amounts of the prepared TiO_2_–SnO_2_ (90–10) wt.% nanocomposite, 0.4 (g/L), photocatalytic degradation of acid dye take place. The observed data showed TiO_2_–SnO_2_ (90–10) wt.% nanocomposite exhibited higher photocatalytic activity than SnO_2_. Additionally, the amount of catalyst utilized increases the rate of dye degradation. The amount of UV photons that the photocatalyst can absorb increases when the loading of the photocatalyst is increased. This caused a rise in the concentration of holes and the excitation of additional electrons from the valence to conduction band. Thus, increasing the number of hydroxyl radicals generated and the photodegradation is enhanced [42].

The superior photocatalytic activity of TiO_2_ over SnO_2_ may be attributed to TiO_2_ smaller particle size, quick (e^−^/h^+^) pair recombination rate, and poor quantum yield of SnO_2_ in aqueous solutions [43].

**Fig. 24:**
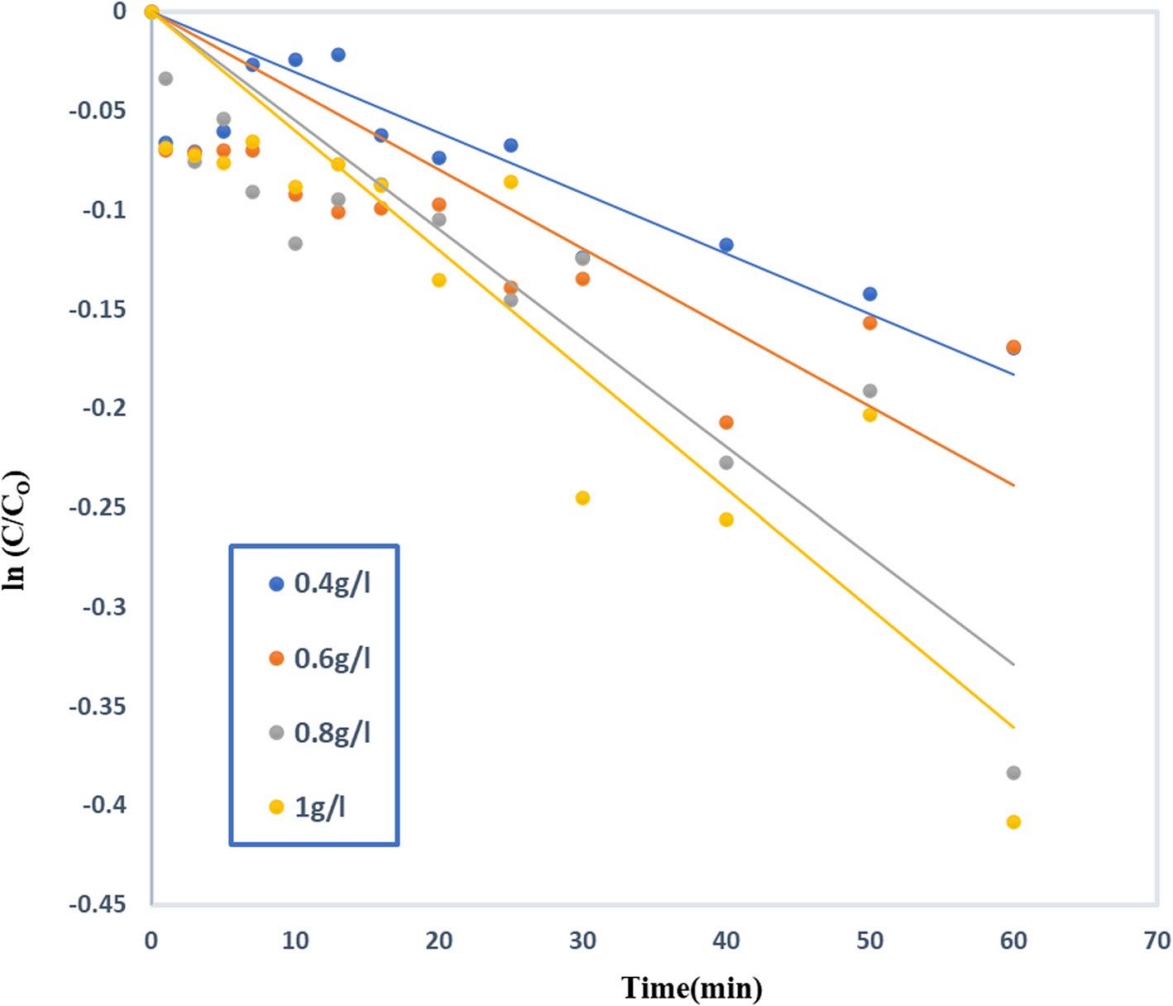
Photocatalytic degradation of acid red 37 (1.0 × 10–4 M) at pH 6.5 using at different loadings of TiO_2_–SnO_2_ (90–10) wt.% (g/L)

**Fig. 25:**
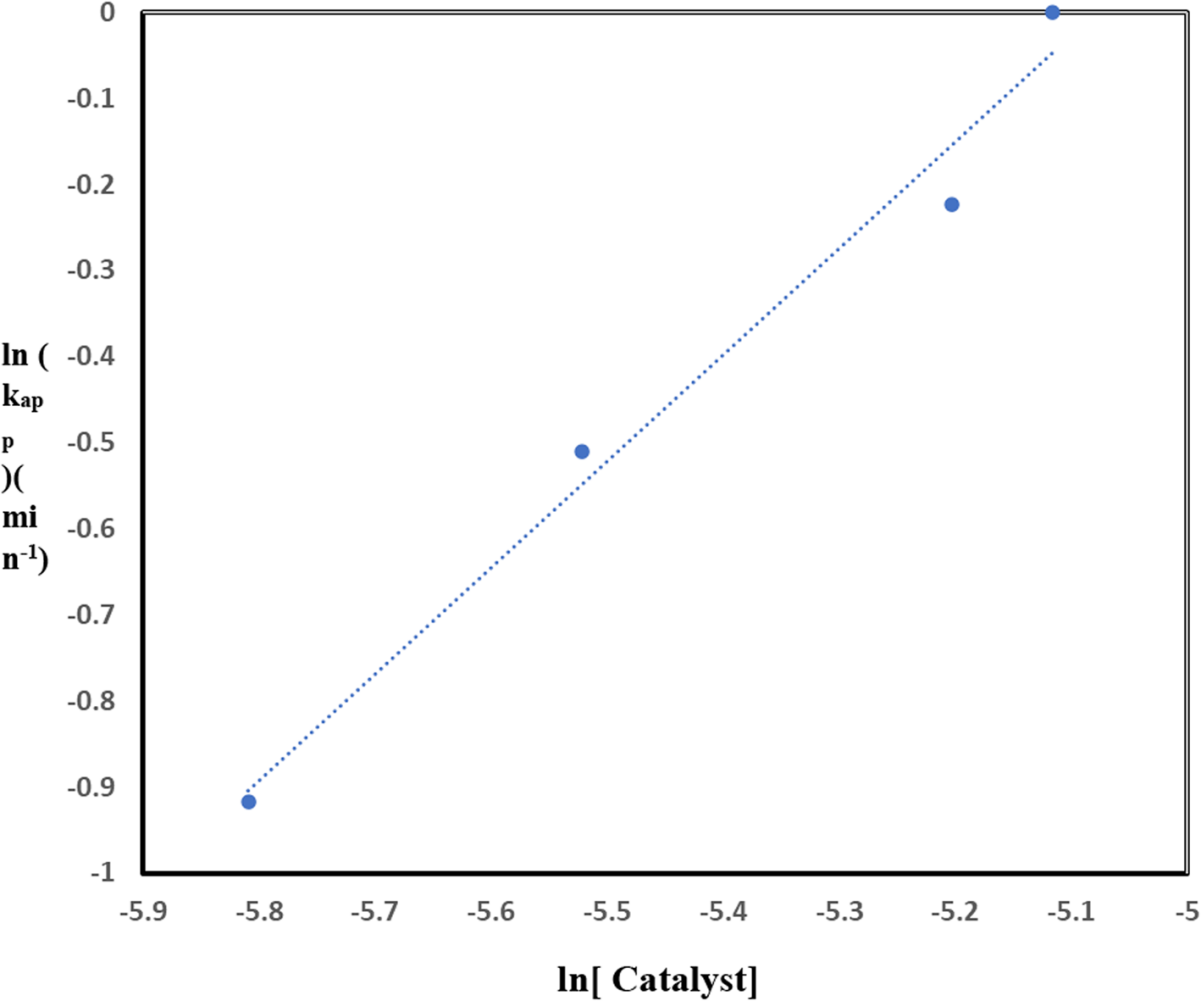
Plot of ln k_app_ (min^−1^) versus ln [Catalyst] for photocatalytic degradation of acid red 37 dye

The catalytic activity of TiO_2_–SnO_2_ (90–10) wt.% nanocomposite was evaluated in the photodegradation of (1.0 × 10^–4^) M of the investigated dye. The nanocomposite with (90:10) showed more photocatalytic activity than that would be possible with only one photocatalyst as in Fig. 24. The apparent reaction orders for the TiO_2_–SnO_2_ (90–10) wt.% nanocomposite were 1.2 as shown in Fig. 25 Table 9, which illustrates the trend of ln k_app_ against ln [catalyst].

**Table 9:** The apparent rate constant and half life time for photocatalytic degradation of acid red 37 dye under UV irradiation using different loading (g/L) TiO_2_–SnO_2_ nanocomposite

Catalytic system	Loading (g/L)	k_app_ (min^−1^)	t_1/2_ (min)	Apparent reaction order (n)
UV/TiO_2_–SnO_2_ (90:10) (g/L)	0.4 g/L	30 × 10^–4^	231	1.2
	0.6 g/L	40 × 10^–4^	173.25	
	0.8 g/L	55 × 10^–4^	126	
	1 g/L	60 × 10^–4^	115.5	

Furthermore, the TiO_2_–SnO_2_ nanocomposite has higher photocatalytic activity than that of TiO_2,_ or SnO_2_ could be related to the more effective separation of photoinduced electron–hole (e^−^/h^+^) pairs due to electron transfer from light-activated SnO_2_'s conduction band.

Table 10 demonstrates the combined data of the apparent rate constant, half life time, and reaction orders for the photocatalytic degradation of acid red 37 dye utilizing several photocatalytic systems UV/TiO_2_, UV/SnO_2_, UV/TiO_2_–SnO_2_ (97:3) wt.%, UV/TiO_2_–SnO_2_ (93:7) wt.%, UV/TiO_2_–SnO_2_ (90:10) wt.%, UV/TiO_2_–SnO_2_ (85:15) wt.%, UV/TiO_2_–SnO_2_ (80:20) wt.%, UV/TiO_2_–SnO_2_ (90:10) wt.%/H_2_O_2_, UV TiO_2_–SnO_2_ (90:10) wt.%/S_2_O_8_ and UV/TiO_2_–SnO_2_ (90:10) wt.%/IO_4_^−^. According to this current study, the three oxidants of peroxide, persulfate, and periodate led to the increase of the rate of acid red 37 dye degradation caused by UV light in the presence of a nanocomposite which was made of TiO_2_–SnO_2_ (90:10) wt.%. The rate of degradation was the fastest when periodate, persulfate, or peroxide was present. Moreover, it was the slowest when UV/TiO_2_ and SnO_2_ were present (90:10) wt.%. It did not appear that the rate enhancing effects of these oxidants had been previously investigated with regard to acid red 37 dye but had been investigated for other organic dyes.

**Table 10:** Collective data of apparent rate constants, half-life times, and reaction orders for degradation of acid red 37 dye

Catalytic system	Concentration	k_app_ (min^−1^)	t_1/2_ (min)	Apparent reaction order (n)
UV/SnO_2_	0.4	2 × 10^–4^	3460	–
UV/TiO_2_	0.4	28 × 10^–4^	247.1	
UV/TiO_2_–SnO_2_ (97:3)	0.4	38 × 10^–4^	182.1	
UV/TiO_2_–SnO_2_ (93:7)	0.4	44 × 10^–4^	157.2	
UV/TiO_2_–SnO_2_ (90:10)	0.4	72 × 10^–4^	96.1	
UV/TiO_2_–SnO_2_ (85:15)	0.4	47 × 10^–4^	147.2	
UV/TiO_2_–SnO_2_ (80:20)	0.4	35 × 10^–4^	197.7	
UV/TiO_2_–SnO_2_ (90:10) (0.4 g/L)/H_2_O_2_	2 × 10^–2^	789 × 10^–4^	8.7	2.3
	3 × 10^–2^	883 × 10^–4^	7.8	
	4 × 10^–2^	943 × 10^–4^	7.3	
	6 × 10^–2^	997 × 10^–4^	6.9	
	8 × 10^–2^	698 × 10^–4^	9.9	
UV/TiO_2_–SnO_2_ (90:10) 0.4 (g/L)/S_2_O_8_^2−^	1 × 10^–3^	166 × 10^–4^	41.7	0.5
	3 × 10^–3^	273 × 10^–4^	25.3	
	5 × 10^–3^	371 × 10^–4^	18.6	
	7 × 10^–3^	826 × 10^–4^	8.3	
	10 × 10^–3^	2352 × 10^–4^	2.9	
UV/TiO_2_–SnO_2_ (90:10) 0.4 (g/L)/IO_4_^−^	0.1 × 10^–3^	251 × 10^–4^	27.6	2.9
	0.15 × 10^–3^	310 × 10^–4^	22.3	
	0.25 × 10^–3^	376 × 10^–4^	18.4	
	0.5 × 10^–3^	638 × 10^–4^	10.8	
	1 × 10^–3^	1214 × 10^–4^	5.7	
Dye 0.1 (g/L), UV/TiO_2_–SnO_2_ (90:10)	0.01 × 10^–3^	861 × 10^–4^	8	1.5
Dye 0.1 (g/L), UV/TiO_2_–SnO_2_ (90:10)	0.03 × 10^–3^	799 × 10^–4^	8.6	
Dye 0.1 (g/L), UV/TiO_2_–SnO_2_ (90:10)	0.05 × 10^–3^	297 × 10^–4^	23.3	
Dye 0.1 (g/L), UV/TiO_2_–SnO_2_ (90:10)	0.07 × 10^–3^	261 × 10^–4^	26.5	
UV/TiO_2_–SnO_2_ (90:10) (pH = 2.0)	0.4 (g/L)	63 × 10^–4^	110	3.3
UV/TiO_2_–SnO_2_ (90:10) (pH = 4.0)	0.4 (g/L)	50 × 10^–4^	138.6	
UV/TiO_2_–SnO_2_ (90:10) (pH = 8.0)	0.4 (g/L)	44 × 10^–4^	157.5	
UV/TiO_2_–SnO_2_ (90:10) (pH = 10)	0.4 (g/L)	38 × 10^–4^	182.3	
Loading (g/L), UV/TiO_2_–SnO_2_ (90:10)	0.4 (g/L)	30 × 10^–4^	231	1.2
Loading (g/L), UV/TiO_2_–SnO_2_ (90:10)	0.6( g/L)	40 × 10^–4^	173.25	
Loading (g/L), UV/TiO_2_–SnO_2_ (90:10)	0.8(g/L) 1(	55 × 10^–4^	126	
Loading (g/L), UV/TiO_2_–SnO_2_ (90:10)	g/L)	60 × 10^–4^	115.5	

## Materials and methods

### Artificial polluted wastewater preparation

**Fig. 26:**
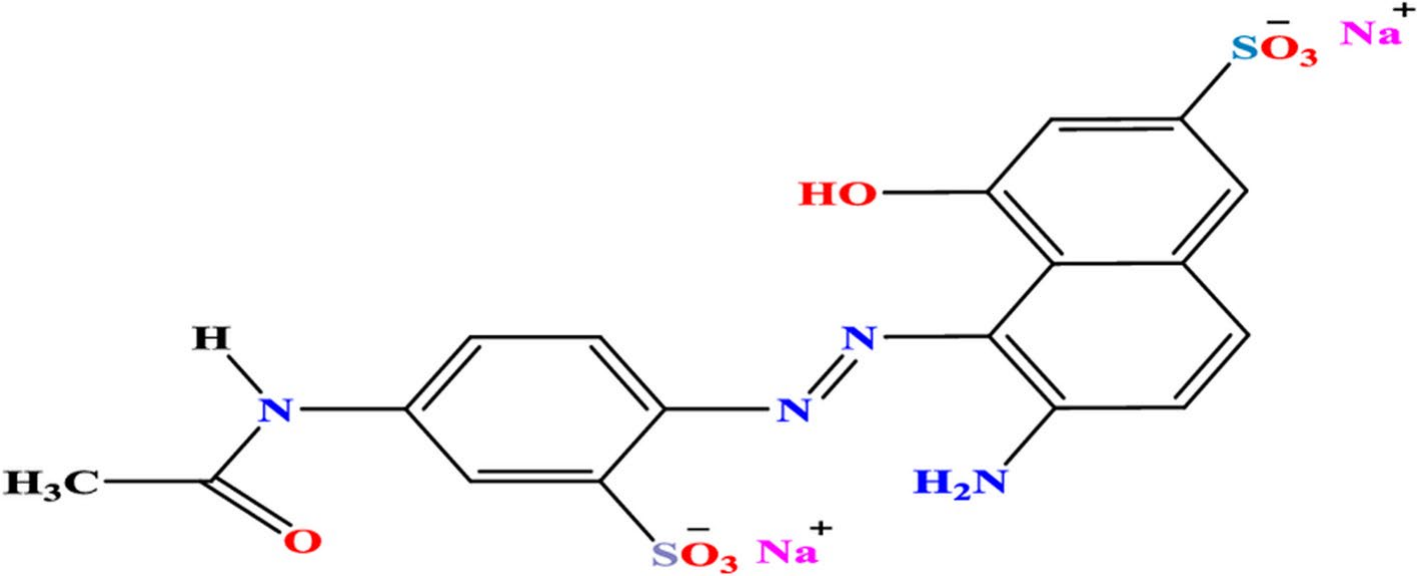
Molecular structure of acid red 37 dye

An aqueous solution of acid red 37 dye (1.04 × 10^−4^) M Fig. 26 was prepared in double distilled water as a model for wastewater pollutant. The pH of TiO_2_–SnO_2_/dye suspension was 6.1. The Addition of H_2_O_2_, NaIO_4_ or Na_2_S_2_O_8_ to TiO_2_–SnO_2_/dye suspension decreased the pH below 6.1. A few drops of an alkali were added to adjust the pH to its original value 6.1.

### Preparation of TiO_2_–SnO_2_ nanocomposites

TiO_2_–SnO_2_ nanocomposites were prepared through a solid-state reaction route. The starting material was TiO_2_ (99.58%). 3, 7, 10, 15 and 20 wt.% of SnO_2_ powder have been added to TiO_2_. The powders have been mixed uniformly by grinding in mortar with pestle for 3 h to get fine powders. The resultant powders have been annealed in air at 500 °C for 3 h in an electric muffle furnace [44].

### Schematic diagram of batch photoreactor

**Fig. 27:**
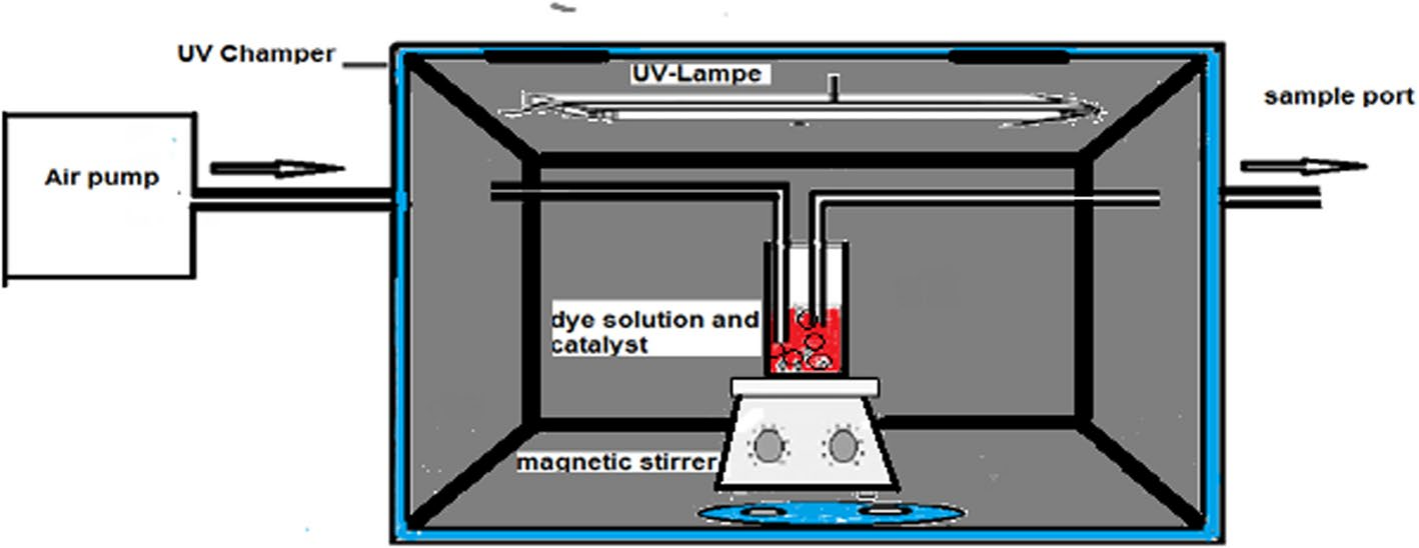
Schematic diagram of the batch photoreactor

The reactor used for examining the photodegradation of the dye throughout experiments was a batch photoreactor (Fig. 27). It consisted of a glass container (250 ml). The contents (TiO_2_/dye, SnO_2_/dye, TiO_2_–SnO_2_/dye, or TiO_2_–SnO_2_/dye/oxidant) of the container were agitated by a magnetic stirrer and kept purged with air at a rate of 3000 ml min^−1^. The dye solution was stirred with TiO_2_–SnO_2_ nanocomposite in the dark for 30 min before irradiation with UV to obtain equilibrium adsorption. Irradiation was carried out with a tubular low-pressure mercury lamp (total rating 43 W, total UV output at 513 nm 13.4 W, and length 120 cm, Voltarc Tubes Inc., USA) and was located 10 cm from the surface of the dye solution. The total intensity reaching the slurry solution was measured using a UVX radiometer (UV Products Ltd., Cambridge) equipped with a sensor with peak sensitivity at 254 nm was 4 mWcm^−2^. Samples of dye solution (1.0 × 10^–4^ mol/L) which had been prepared in distilled water as a model for wastewater pollutants were removed from the container via a sample port periodically and measured after filtration through 0.2 µm polyether sulfone membrane. All photodegradation experiments were done in a temperature of 22 ± 2 °C.

### Absorbance measurements

The color fading of the dye was analyzed spectrophotometrically at its maximum absorption wavelength of 513 nm, using Shimadzu Model 1601 PC double beam spectrophotometer. Samples containing TiO_2_–SnO_2_ nanopowder were taken periodically from the photoreactor and measured after filtration using 0.2 µm polythersulfone membrane.

### Measurement of photocatalytic activity

The photocatalytic performance of native TiO_2_, SnO_2_ nanoparticles and TiO_2_–SnO_2_ nanocomposites was investigated by the decomposition of acid red 37 dye in an aqueous solution under UV light (254 nm). Absorbance was readily described by Beer–Lambert law [45].

13 $$ {\text{A}} = \varepsilon {\text{LC}} $$

where A is the absorbance, ε is the molar extinction coefficient, L is the light path length (1.00 cm), and C is the concentration. The absorbance which had been given at each irradiation time was related to a specific dye concentration using a calibration curve for acid red 37 dye solutions with different concentrations. The percentage of degradation of the dye could be calculated according to Eq. (3):

14 $$ {\text{Degradation }}\% = {\text{ C}}_{{\text{o}}} - {\text{ C}}/{\text{C}}_{{\text{o}}} \; \times \; 100 $$

where C_o_ = initial concentration of dye solution, C = concentration of dye solution after photoirradiation at time t.

## Conclusions

The value for SnO_2_ was determined to be lower than that of TiO_2_. However, the TiO_2_–SnO_2_ composite had demonstrated to have much higher photocatalytic activity as compared to both the native SnO_2_ and the native TiO_2_. Consequently, it was determined that the ratio of UV/TiO_2_–SnO_2_ (90:10) wt.% nanocomposite was having higher efficiency photocatalytic degradation from the other ratios when ratios were examined through characterization by XRD, TEM, EDAX, FTIR, and XPS. The heterogeneous photocatalytic degradation of the examined acid red 37 dye with a TiO_2_–SnO_2_ nanocomposite at several (3–20) wt.% had demonstrated that the UV/TiO_2_–SnO_2_ (90:10) wt.% had been exhibiting superior photocatalytic degradation of the acid red 37 dye in comparison to the other ratios. Furthermore, the following order was established with the addition of oxidants (H_2_O_2_, S_2_O_8_^2−^, or IO_4_^−^) where being expressed as: UV/TiO_2_–SnO_2_ (90:10) wt.%/IO_4_^−^ were having higher efficiency photocatalytic degradation of acid red 37 dye in wastewater treatment than from other oxidants. Finally, as the pH was decreasing, the rate of degradation was increasing. On the other hand, where the dye concentration was increasing, the degradation was increasing, as well. By the same token, as the loading of photocatalyst was increasing, the rate of degradation was rising.

## Data Availability

All data are available upon reasonable request.
